# The SMC-5/6 Complex and the HIM-6 (BLM) Helicase Synergistically Promote Meiotic Recombination Intermediate Processing and Chromosome Maturation during *Caenorhabditis elegans* Meiosis

**DOI:** 10.1371/journal.pgen.1005872

**Published:** 2016-03-24

**Authors:** Ye Hong, Remi Sonneville, Ana Agostinho, Bettina Meier, Bin Wang, J. Julian Blow, Anton Gartner

**Affiliations:** Centre for Gene Regulation and Expression, University of Dundee, Dundee, United Kingdom; The University of North Carolina at Chapel Hill, UNITED STATES

## Abstract

Meiotic recombination is essential for the repair of programmed double strand breaks (DSBs) to generate crossovers (COs) during meiosis. The efficient processing of meiotic recombination intermediates not only needs various resolvases but also requires proper meiotic chromosome structure. The Smc5/6 complex belongs to the structural maintenance of chromosome (SMC) family and is closely related to cohesin and condensin. Although the Smc5/6 complex has been implicated in the processing of recombination intermediates during meiosis, it is not known how Smc5/6 controls meiotic DSB repair. Here, using *Caenorhabditis elegans* we show that the SMC-5/6 complex acts synergistically with HIM-6, an ortholog of the human Bloom syndrome helicase (BLM) during meiotic recombination. The concerted action of the SMC-5/6 complex and HIM-6 is important for processing recombination intermediates, CO regulation and bivalent maturation. Careful examination of meiotic chromosomal morphology reveals an accumulation of inter-chromosomal bridges in *smc-5; him-6* double mutants, leading to compromised chromosome segregation during meiotic cell divisions. Interestingly, we found that the lethality of *smc-5; him-6* can be rescued by loss of the conserved BRCA1 ortholog BRC-1. Furthermore, the combined deletion of *smc-5* and *him-6* leads to an irregular distribution of condensin and to chromosome decondensation defects reminiscent of condensin depletion. Lethality conferred by condensin depletion can also be rescued by BRC-1 depletion. Our results suggest that SMC-5/6 and HIM-6 can synergistically regulate recombination intermediate metabolism and suppress ectopic recombination by controlling chromosome architecture during meiosis.

## Author Summary

Meiosis is a special form of cell division needed for the formation of haploid gametes. During meiosis, DNA double-strand breaks are enzymatically induced and then repaired by the meiotic recombination pathway. Meiotic recombination is essential for genetic diversity and for accurate segregation of chromosomes during meiosis. Meiotic recombinational repair can either use the homolog or the sister chromatid as a template to generate different recombination intermediates. Recombination intermediates must be resolved or dissolved by specific endonucleases and the BLM helicase. Unresolved recombination intermediates lead to formation of chromatin bridges and perturb proper chromosome segregation. In this study we show that the concerted action of the BLM helicase HIM-6 and the evolutionarily conserved SMC-5/6 complex are important for the ordered processing of meiotic recombination intermediates, proper crossover (CO) formation and subsequent chromosome segregation in *C*. *elegans*. Notably, HIM-6 is required for the normal distribution of meiotic COs. We also propose that the interplay between SMC-5/6 and HIM-6 has a crucial role in the formation of a highly condensed and ordered chromosomal structure, which constrains BRC-1 dependent ectopic recombination.

## Introduction

Homologous recombination (HR) accurately repairs DNA double-strand breaks (DSBs). During recombination, DSBs are resected to create long single-strand DNA tails, which can invade intact homologous donor sequences with the aid of the conserved Rad51 recombinase. An initial recombination intermediate formed after strand invasion is called a displacement loop (D-loop). The invading strand then primes DNA synthesis using the intact homologous chromosomes as a template and second-end DNA capture leads to formation of cruciform recombination intermediates known as Holliday junctions (HJs). HJs can be dissolved by BLM helicase combined with topoisomerase III and RMI1 [1] or resolved by different structure-specific endonucleases leading to crossover (CO) or non-crossover (non-CO) products.

Homologous recombination is essential for meiosis [2]. Meiotic DSBs are generated by the Spo11 enzyme [3]. The choice of template for repairing programmed DSBs during meiosis is distinct from that during mitosis [4]. In mitotic cells, the sister chromatids are preferentially used as repair templates, thereby preventing potentially deleterious effects of recombination, such as loss of heterozygosity. In contrast, during meiosis there is a bias to use the homolog as a repair template, therefore facilitating the formation of COs between homologous chromosomes [5]. COs are not only important for the exchange of genetic information between maternal and paternal chromosomes, but together with sister chromatid cohesion they also provide a transient physical linkage between homologs (chiasmata) that prevents precocious chromosomes segregation before the end of meiosis I.

Although interhomolog recombination is generally thought to prevail during meiosis, intersister recombination also plays an important role in meiotic DSB repair (for review see [6]). In budding yeast, DSBs are efficiently repaired by intersister recombination during meiosis, even when the homolog is present [7]. In *C*. *elegans*, interhomolog recombination is favored to repair SPO-11-induced DSBs during the early and mid-pachytene stage [8]. In contrast, intersister recombination contributes to rapid DNA repair at the late pachytene stage in order to preserve genomic integrity before exit from meiotic prophase [9, 10].

At the end of the meiotic prophase, all the recombination intermediates generated by interhomolog and intersister recombination must be resolved to facilitate CO formation and accurate chromosome segregation. Numerous studies have been done to identify HJ resolvases contributing to CO formation in different organisms [11–14]. In budding yeast, at least five endonucleases, belonging to two distinct pathways, are required for CO formation during meiosis [12, 15]. The major pathway including Exo1 and the Mlh1-Mlh3 (MutLγ) complex produces the majority of COs while the minor pathway involves the Mus81-Mms4, Yen1, and Slx1-Slx4 complex. Although these enzymes (except for Mlh3) are conserved in *C*. *elegans*, different sets of resolvases appear to be used [16–18]. Based on genetic and cytological data, *C*. *elegans* might have at least two redundant resolvase activities for meiotic recombination intermediate resolution and CO formation. The first resolvase activity depends on XPF-1 and BLM helicase HIM-6. The other resolvase activity includes SLX-1 and MUS-81. Deletion of both resolvase activities results in severely reduced progeny viability and persistence of chromatin bridges between homologs, which represent unresolved recombination intermediates [16]. However, few wild type COs are still present even in the absence of both resolvase activities, and the *mus-81 slx-1; xpf-1; him-6* quadruple mutant is not 100% lethal, suggesting the existence of further factors capable of resolving HJs [16].

The Smc5/6 complex belongs to the structure maintenance of chromosome (SMC) protein family, which also includes components of cohesin and condensin and is well conserved among eukaryotes [19–21]. Smc5 and Smc6 form a heterodimeric ring-like structure via the interaction through their hinge domains [22]. In budding yeast, at least six additional subunits are associated to Smc5 and Smc6. These subunits are termed non-SMC elements (Nse1–6) [23], whereas in human and worms only four Nse subunits have been identified so far [24]. Notably, Nse1 contains a RING finger domain that resembles those found in ubiquitin ligases and the C-terminal portion of Nse2 contains a conserved RING-like domain and shows SUMO-ligase activity both *in vitro* and *in vivo* [25, 26]. While the function of cohesin and condensin in sister chromatid cohesion and chromosome condensation has been well characterized, little is known about the exact cellular function of Smc5/6. Previous studies show that the Smc5/6 complex is required for recombinational repair in mitotic cells [27, 28]. Recently, the Smc5/6 complex has been found to locate at the pericentromeric heterochromatin in mouse spermatocytes, indicating that it might be involved in preventing aberrant HR in these repetitive regions during meiosis [29, 30]. However, this localization is not conserved in human prophase spermatocytes [31]. The Smc5/6 complex is also important for the elimination of meiotic recombination intermediates in budding yeast [32–34]. Notably, budding yeast Smc5/6 are essential for cell viability while genes involved in homologous recombination are not, suggesting that the compromised DNA repair in smc5/6 mutants might also originate from a more fundamental defect in chromosome organization. However, the cross-talk between the Smc5/6 complex and other SMC complexes remains elusive. While a regulatory role of the Smc5/6 complex in Mus81-Eme1 dependent HJ resolution has been revealed in fission yeast [35], the exact role of Smc5/6 in higher organisms and the interplay between Smc5/6 complex and other meiotic recombination intermediate resolvases, such as the BLM helicase, is largely unknown.

The hereditary breast/ovarian cancer predisposition gene BRCA1, which is not present in budding and fission yeast, forms a heterodimer with BARD1 and is evolutionarily conserved. The BRCA1/BARD1 complex has been reported to be involved in a variety of processes in somatic cells, including DNA replication, DNA damage response and chromatin remodeling [36]. BRCA1 has also been suggested to have a role in meiotic DSB repair. Previous studies showed that BRCA1 interacts with RAD51 and that it is required for loading of RAD51 to DSB sites [37]. In contrast, a recent study suggested that BRCA1 has a modest impact on RAD51 assembly and CO formation [38]. While deletion of *Brca1* exon 11 disrupts spermatogenesis in mice, *Brca1*^*Δ11/Δ11*^ female mice are fertile [39]. However, *Brca1* mutants have a decreased number of MSH4 foci, which is thought to be associated with the stabilization of single-strand invasion intermediates formed at early stages of recombination. This indicates a role of BRCA1 in the regulation of recombination intermediates [38]. Mutants of the *C*. *elegans* BRCA1 ortholog *brc-1* are also viable and fertile but display a weak meiotic chromosome segregation defect [10]. However, when interhomolog recombination is abrogated by depletion of the synaptonemal complex component SYP-2, mutation of *brc-1* results in significantly increased chromosome fragmentation, suggesting that BRC-1 is important for meiotic DSB repair through intersister recombination [10].

In this study, we show that SMC-5/6 acts synergistically with the BLM helicase HIM-6 in meiotic DSB repair in *C*. *elegans*. The combined function of SMC-5/6 and HIM-6 is essential for recombination intermediate processing, CO formation and accurate chromosome segregation during meiosis. Loss of BRC-1 rescued the progeny lethality of *smc-5; him-6* double mutants. Furthermore, deletion of SMC-5/6 and HIM-6 leads to an irregular distribution of condensin and defective chromosome morphology. Our results suggest that SMC-5/6 and HIM-6 can synergistically regulate recombination intermediate metabolism and prevent ectopic recombination by controlling chromosome architecture during meiosis.

## Results

### SMC-5/6 and HIM-6 act synergistically to prevent accumulation of recombination intermediates during meiosis

**Fig 1:**
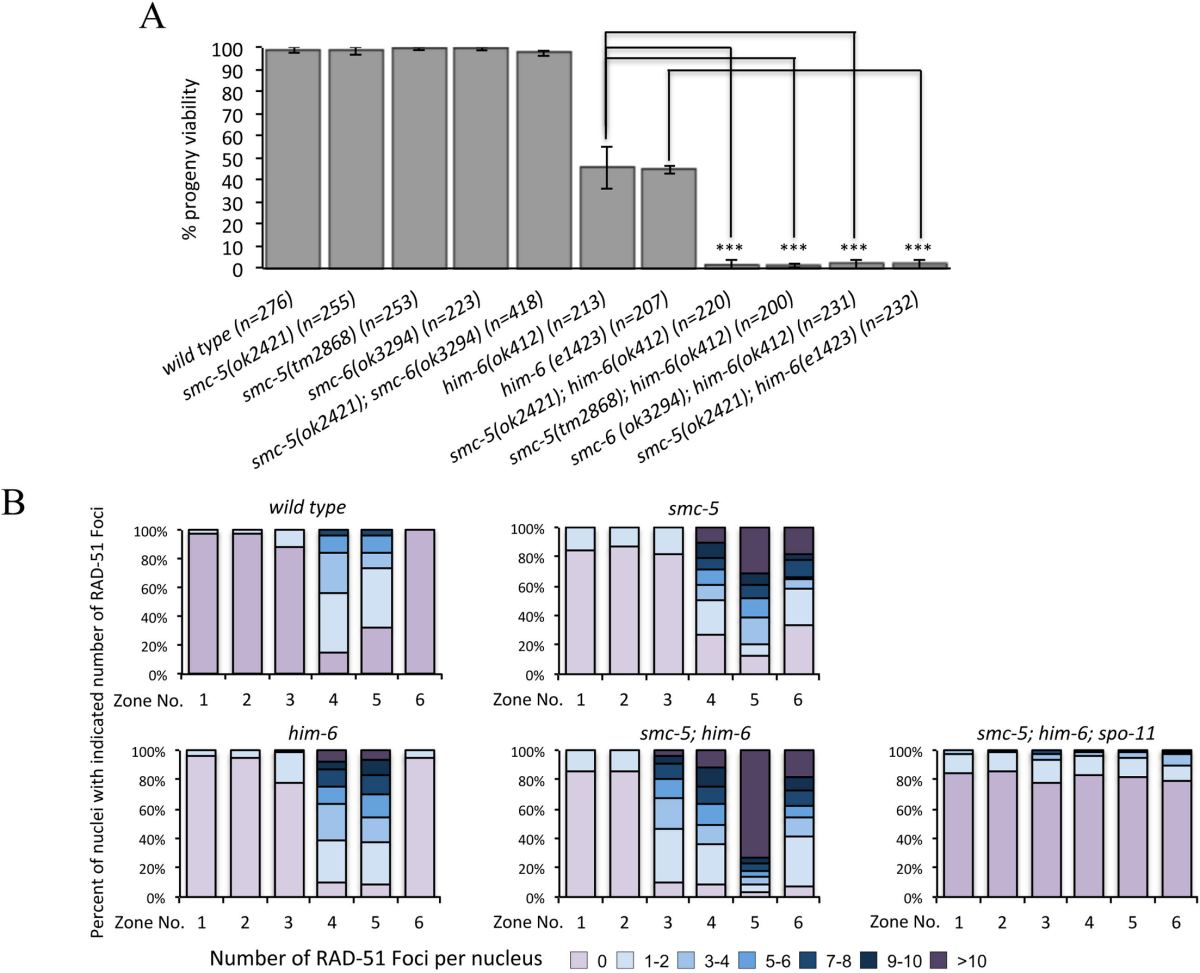
Synergistic function of SMC-5/6 and the BLM helicase HIM-6. **A.** Genetic interaction between SMC-5/6 complex and HIM-6 was examined by combining *smc-5/6* deletion with different *him-6* alleles. Progeny viability in % was determined by counting number of viable eggs/total number of eggs laid. The sample size (n) indicates the number of embryos examined for each genotype. Error bars represent standard deviation of the mean. Asterisks indicate statistically significant reduction in embryonic viability in *smc-5(ok2421); him-6(ok412)*, *smc-5(tm2868); him-6(ok412)*, *smc-5(ok2421); him-6(e1423)* and *smc-6(ok3294); him-6(ok412)* double mutants (p < 0.005 by two-tailed Student T-test) when compared with *him-6(ok412)* and *him-6(e1423)*. **B.** Distribution of RAD-51 foci in wild type, *smc-5(ok2421)*, *him-6(ok412)*, and *smc-5(ok2421); him-6(ok412)* animals. Zone definitions: 1 Early mitotic, 2 Late mitotic, 3 Transition, 4 Early pachytene, 5 Middle pachytene, 6 Late pachytene.

The BLM helicase is a central regulator of meiotic recombination and is essential for meiotic HJ resolution and CO formation [12, 15, 40]. To study the interplay between the SMC-5/6 complex and the BLM helicase HIM-6 during meiotic DNA repair in *C*. *elegans*, we examined the effects of *smc-5/6* deletion in conjunction with various *him-6* alleles. As previously reported, the strong alleles *ok412* and *e1423* abrogate the HIM-6 helicase domain and cause a severe reduction of progeny viability (46% and 44% viable embryos, respectively) [16, 41, 42]. In contrast, the progeny viability of *smc-5(ok2421)*, *smc-5(tm2868)* and *smc-6(ok3294)* single mutants and the *smc-5(ok2421); smc-6(ok3294)* double mutant was similar to that of the wild type (about 99%), suggesting that SMC-5/6 complex is dispensable for viability in *C*. *elegans*, consistent with a previous study (Fig 1A) [43]. We found that both *smc-5* and *smc-6* were synthetic lethal with *him-6* (Fig 1A). The progeny viability is dramatically decreased in *smc-5(ok2421); him-6(ok412)* and *smc-5(ok2421); him-6(e1423)* as well as in *smc-6(ok3294); him-6(ok412)* double mutants (1–2% viable embryos, p<0.005 in all cases) (Fig 1A).

To assess if the synthetic lethality we observed was due to a defect in recombinational repair, we examined the appearance and disappearance of the strand exchange protein RAD-51 on meiotic chromosomes. RAD-51 binds to the resected single strands and the number of RAD-51 foci provides an estimate of the number of recombination intermediates [44]. Increased RAD-51 foci formation is typically observed in the transition zone, a stage where meiotic DSBs are induced (Fig 1B, Zone 3, S1 Fig). As recombination progresses, all DSBs are repaired and RAD-51 foci finally disappear at the late pachytene stage (Fig 1B, zone 6, S1 Fig). Consistent with previous studies, a significantly increased number of RAD-51 foci were observed in early and mid pachytene zones in the *smc-5* and *him-6* single mutants when compared to the wild type animals [17, 41, 43]. While the RAD-51 staining was rarely seen in the late pachytene in wild type and *him-6* mutants, it persisted in *smc-5* mutants as reported previously [43]. Notably, in the *smc-5(ok2421); him-6(ok412)* double mutants more RAD-51 foci were detected at all meiotic zones compared to either single mutant (Figs 1B and S1). We did not observe a dramatically elevated number of RAD-51 foci in mitotic germ cells (Fig 1B, zone 1 and 2, S1 Fig) of *smc-5(ok2421); him-6(ok412)*, and the elevated number of RAD-51 foci depends on SPO-11 (Figs 1 and S1), confirming that SMC-5/6 and HIM-6 cooperate to process meiotic SPO-11 dependent DSBs.

### SMC-5/6 and HIM-6 are dispensable for meiotic chromosome axis formation and synapsis

Compromised meiotic DSB repair can be due to a defect in the establishment of early meiotic events, such as chromosome axis formation and synapsis [45]. To test whether these events are defective in *smc-5(ok2421); him-6(ok412)*, we analyzed the localization of HTP-3, a component of the *C*. *elegans* axial element, and SYP-1, a component of the synaptonemal complex (SC) central region. Axial element HTP-3 coordinates meiotic DSB formation, homologous pairing and synapsis and localizes along the length of parallel DAPI tracks in pachytene stage [46]. SYP-1 is a component of the central region of the synaptonemal complex [47]. We found that the localization of HTP-3 and SYP-1 occurs normally in *smc-5(ok2421) and him-6(ok412)* single mutants and *smc-5(ok2421); him-6(ok412)* double mutants during pachytene by immunostaining (S2 Fig). We therefore conclude that SMC-5/6 and HIM-6 are dispensable for chromosome axis and SC formation.

### Loss of SMC-5/6 and HIM-6 affects the maturation of meiotic bivalents

**Fig 2:**
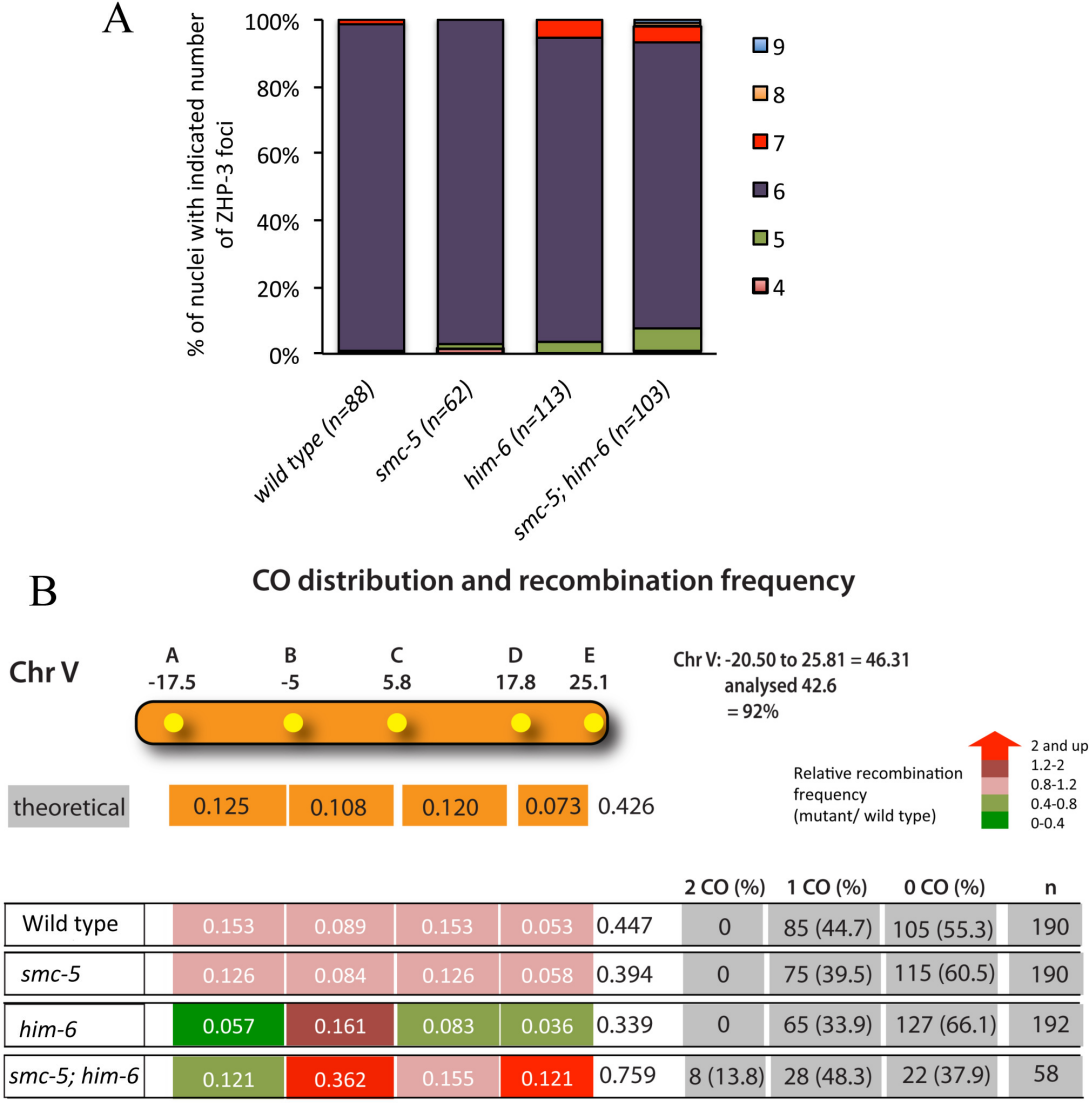
SMC-5/6 complex and HIM-6 are required for the regulation of meiotic crossover formation. **A.** Quantification of ZHP-3::GFP foci in pachytene nuclei of wild type, *smc-5(ok2421)*, *him-6(ok412)*, and *smc-5(ok2421); him-6(ok412)* mutants. The sample size number (n) indicates the number of germ nuclei examined for each genotype. **B**. Analysis of CO frequencies and distribution on chromosome V. The genetic map positions of the five SNPs, which together cover 92% of chromosome V, are indicated. n is the number of cross-progeny scored. The frequency of 2 COs, 1 CO or 0 CO per chromosome is indicated in absolute numbers and as percentage (in brackets). The relative recombination frequencies (mutant/ wild type) are indicated by different coloured tags. Red reflects the greatest increase and green reflects the greatest decrease.

To investigate whether the SMC-5/6 complex and HIM-6 are required for late meiotic CO formation, we examined CO designation in *smc-5(ok2421); him-6(ok412)*, as well as in the corresponding single mutants. ZHP-3 has been proposed to mark meiotic DSBs designated for CO recombination [48, 49]. In the wild type, ZHP-3 initially localizes along the length of chromosomes, but becomes restricted to six distinct foci per nucleus in late pachytene stage, indicating one CO precursor per bivalent. The average number of ZHP-3 foci remained unchanged at the wild type level of ~six foci per nucleus in the *smc-5(ok2421)* (5.95 per nucleus) and *him-6(ok412)* (6.02 per nucleus) single mutant germ cells (Fig 2A), in accordance with previous studies [16, 43]. In *smc-5(ok2421); him-6(ok412)* double mutants, most nuclei (>86%) have six ZHP-3 foci (~6.01 per nucleus, p = 0.482 when compared to *him-6*, p<0.05 as significant), indicating that the CO designation is not compromised in *smc-5(ok2421); him-6(ok412)* double mutants.

We next directly investigated whether the CO frequency and distribution are defective in *smc-5(ok2421); him-6(ok412)* double mutant employing Snip-SNPs (single nucleotide polymorphisms) to differentiate between N2 and polymorphic Hawaii chromosomes [50]. We generated the respective single and double mutants with chromosome V being heterozygous for Hawaii and N2. To investigate the recombination frequency and distribution we used five snip-SNPs, which together cover 92% of chromosome V [51]. It is known that COs are enriched at the arm regions of the autosomes in *C*. *elegans* and are suppressed at the center of the chromosomes [52, 53]. In the wild type, 44.7% of chromatids have a single CO, and no double COs could be detected. *smc-5(ok2421)* mutants did not show an altered CO frequency or distribution (Fig 2B). Nevertheless, consistent with previous studies, *him-*6*(ok412)* shows a reduced recombination rate in general, with 33.9% of single CO chromatids [16, 41, 54, 55] (Fig 2B). However, although the frequency of COs was dramatically decreased in the arm regions of chromosome V in *him-6(ok412)* mutants, it was 1.8 fold higher in the central region which we defined as between -5 and +5.8 and comprises ~one-fourth of the chromosome V. We further confirmed the occurrence of a higher CO frequency (~2-fold) at the central region of chromosome I in *him-6(ok412)* mutants (S3 Fig), consistent with a previous study [55]. This result is also supported by a recent report showing that the HIM-6 interacting protein RMH-1 (RMI1 homolog) antagonizes CO formation in the center of chromosomes [56]. Interestingly, when *him-6* mutation combined with *smc-5*, CO frequency was enhanced, the strongest enhancement occurring at the center of the chromosome (Fig 2B). In addition, in *smc-5(ok2421); him-6(ok412)* a significant frequency of double COs was also detectable (13.8%), indicating that CO interference may be impaired in the absence of both SMC-5/6 complex and HIM-6 (Fig 2B).

**Fig 3:**
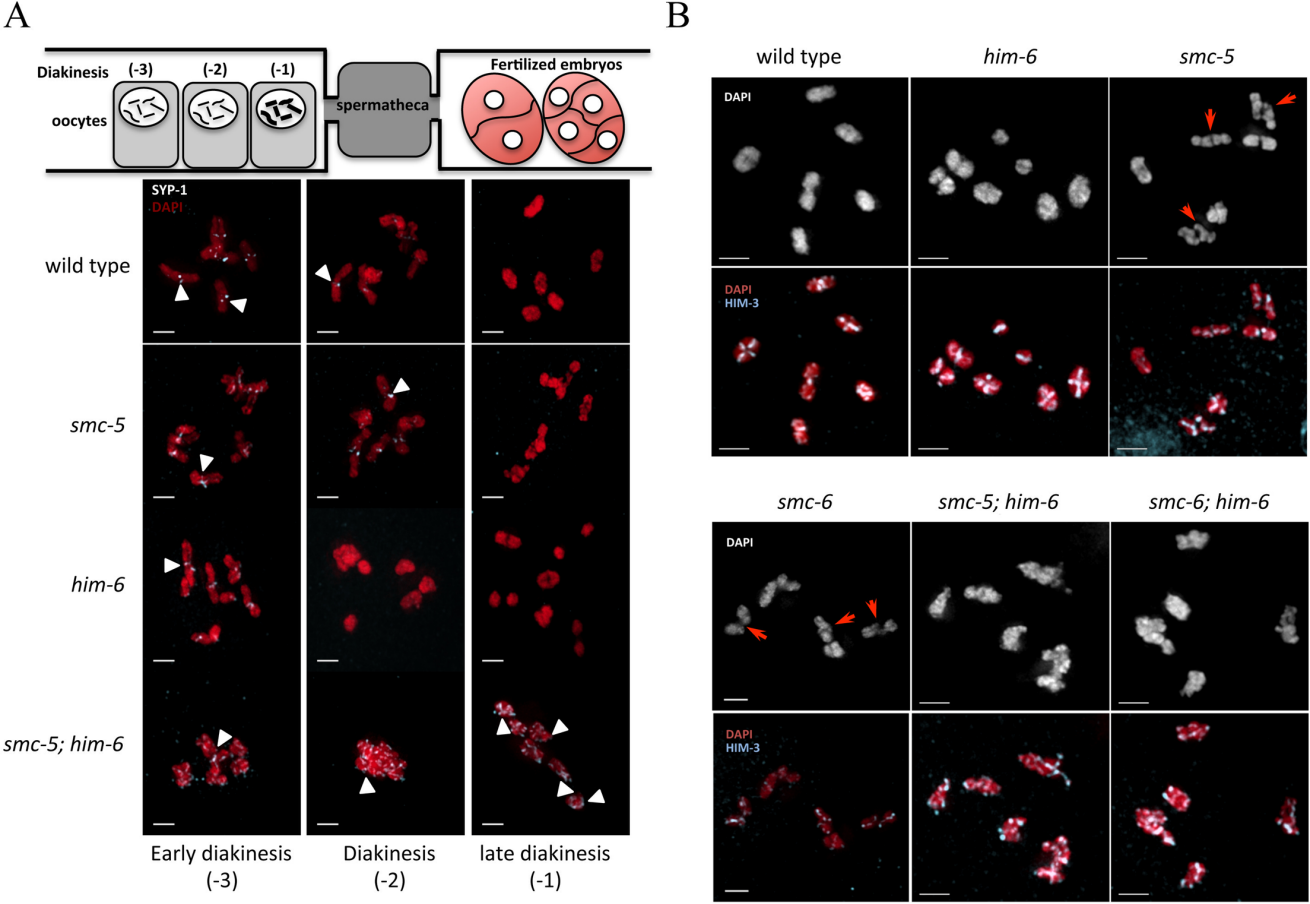
SMC-5/6 and HIM-6 are required for the maturation of meiotic chromosomes. **A.** SYP-1 immunostaining of representative diakinesis nuclei of wild type, *smc-5(ok2421)*, *him-6(ok412)*, and *smc-5(ok2421); him-6(ok412)* mutants. White arrowheads indicate the remaining SYP-1 staining. **B.** Representative images of nuclei of diakinesis oocytes stained with an antibody recognizing the chromosome axis component HIM-3. Red arrows indicate the not well-compacted bivalents. Scale bars: 2 μm.

CO formation can also be analyzed cytologically by examining the maturation of bivalents in the diakinesis stage of meiosis I. In wild type, the establishment of COs is accompanied by the differentiation of bivalents into functionally distinct short and long arms demarked by the CO site. In early diakinesis, synaptonemal complex proteins such as SYP-1/2 are concentrated on short arm of the bivalent. In late diakinesis, SYP-1/2 disappears as bivalents mature [57]. While SYP-1 could hardly be detected in the -2 oocytes (the next most proximal oocyte, position relative to spermatheca, Fig 3A) in wild type and *him-6* mutants, most bivalents still exhibited SYP-1 staining in -2 oocytes in *smc-5* mutants. However, no visible SYP-1 remained in -1 oocytes (the most proximal oocyte) in all single mutants and wild type animals (Fig 3A). In contrast, in *smc-5(ok2421); him-6(ok412)* double mutants, SYP-1 staining persisted in all -1 oocytes observed (Fig 3A arrowheads). Furthermore, bivalent maturation can be assessed by staining the axial element component HIM-3 [58]. In nuclei from wild type and *him-6(ok412)* single mutants, bivalents were highly condensed and appeared as compact DAPI-staining bodies with a cruciform HIM-3 staining pattern indicative of normal chiasma formation (Fig 3B). In contrast, some bivalents in *smc-5(ok2421)* and *smc-6(ok3294)* single mutants were not well resolved as previously reported (Fig 3B) [43]. Affected bivalents had an elongated chromosome axis (Fig 3B, red arrow, for high resolution S4 Fig). Intriguingly, the bivalents in *smc-5(ok2421); him-6(ok412)* double mutants were poorly condensed, HIM-3 staining was disorganized and no cruciform structure could be observed, indicating a defect in chiasmata formation at the diakinesis stage (Fig 3B).

### Abrogation of SMC-5/6 and HIM-6 leads to formation of chromatin bridges between meiotic chromosomes

**Fig 4:**
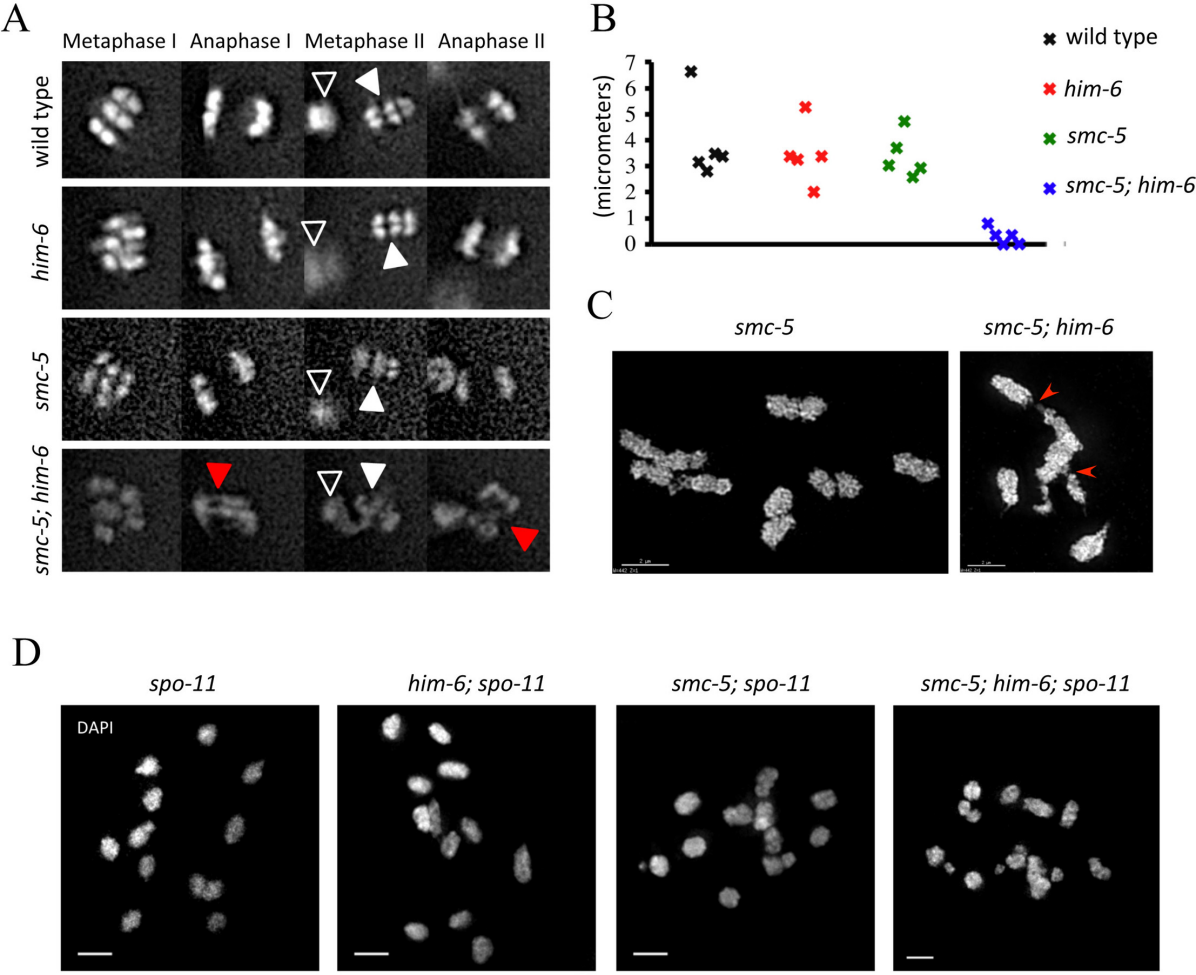
Compromised chromosome segregation caused by chromatin linkages during meiotic division. **A.** Representative images taken from time-lapse recordings of GFP-Histone H2B expressing embryos during meiotic division. Black arrowheads indicate the first polar body; white arrowheads indicate chromosomes aligned on the metaphase plate. Red arrowheads indicate the chromatin linkages. **B.** Graph depicting the distance between the first polar body and the metaphase plate one minute prior to the onset of anaphase II. The distances in *smc-5; him-6* double mutants (0.29±0.32 μm) were significant different from the wild type (3.89±1.55 μm), *him-6* (3.46±1.16 μm) and *smc-5* (3.39±0.85) single mutants (p<0.001). Statistical significance was determined by two-tailed Student T-test. P Values below 0.05 were considered significant. A minimum of five embryos were analysed for each genotype. **C.** Representative OMX images of diakinesis nuclei of *smc-5(ok2421)* and *smc-5(ok2421); him-6(ok412)* mutants. Red arrowheads indicate the chromatin linkages. **D**. Images of DAPI-stained chromosomes in –1 oocytes at diakinesis in the indicated genotypes. Scale bars: 2 μm.

It is known that proper recombination intermediate processing and CO formation are required for chromosome segregation [16, 32, 59]. Because *smc-5(ok2421); him-6(ok412)* double mutants have an increased number of recombination intermediates as evidenced by RAD-51 staining, compared to *smc-5(ok2421)* and *him-6(ok412)* single mutants, we sought to examine whether chromosome segregation was impaired by chromatin bridges using live cell imaging of meiotic cell divisions. In wild type and *him-6(ok412)* or *smc-5(ok2421)* single mutants, meiotic chromosomes segregated with no detectable chromatin bridges (Fig 4A, S1–S3 Videos). In contrast, in *smc-5(ok2421); him-6(ok412)* double mutants chromosomes were linked by chromatin bridges during anaphase I and II and could not separate properly (Fig 4A, S4 Video). Measurements of the distance between the metaphase plate and the polar body during the first meiotic division in different mutants revealed that there is no difference between wild type and *smc-5* (p = 0.6575) or *him-6* (p = 0.5428) single mutants, but the distance was dramatically decreased in *smc-5(ok2421); him-6(ok412)* double mutants (p = 0.00093, <0.001). In summary, our analysis shows that both homologs (meiosis I) and sister chromatids (meiosis II) are connected by chromatin bridges in *smc-5(ok2421); him-6(ok412)* double mutants (Fig 4A and 4B, S4 Video). This finding is consistent with recent observation that SMC-5 and RMH-1 cooperate to prevent accumulation of aberrant chromosome connections [56].

We next carefully examined diakinesis chromosomes from different mutants using super resolution microscopy. While wild type had six bivalents per oocyte and no univalent can be detected, most of the oocytes (88%) from *him-6(ok412)* mutants we examined had some univalents as indicated by more than six DAPI staining bodies (S5 Fig for quantification). In contrast, no univalent could be observed in *smc-5(ok2421); him-6(ok412)* double mutants, and 53% of oocytes have less than six DAPI stained bodies, bivalents linked by chromosome bridges being scored by us as one (Fig 4C, red arrow for chromatin bridge, S3 Fig for quantification, S5 Video, see linked bivalent at the bottom left). *smc-5(ok2421)* mutants occasionally contained linked bivalents (S5 Fig for quantification).

The Smc5/6 complex and the BLM helicase have been implicated in DNA repair in mitotically dividing cells [60, 61]. Thus, the chromatin bridges could represent unresolved DNA repair intermediates, possibly carried over from mitotic cell divisions. Alternatively, the bridges we observed might originate from SPO-11 induced DSBs, and represent unresolved meiotic recombination intermediates. To distinguish between these possibilities, we generated *smc-5(ok2421); him-6(ok412); spo-11* triple mutants. Loss of SPO-11 abolishes meiotic recombination, which leads to presence of twelve univalents in diakinesis oocytes due to the absence of chiasma between homolog pairs. In *smc-5(ok2421); him-6(ok412); spo-11* triple mutants, we rarely observed chromosome bridges between univalents. Although chromosome fragments, which likely originated from defects in mitotic DNA repair, could be detected, the number of DAPI-stained bodies didn’t change significantly (11.9±1.37, N = 20) (Fig 4D). We thus conclude that the chromatin bridges are largely derived from SPO-11 induced unresolved meiotic recombination intermediates.

In mutants defective for HR, deletions and translocations can arise due to the error-prone repair of DSBs by non-homolog end joining (NHEJ) [62–64]. DSB repair by NHEJ involves the direct re-ligation of broken DNA ends and requires Ku proteins, ligase IV, and a number of other factors [65]. To determine whether the chromatin bridges that occur in *smc-5(ok2421); him-6(ok412)* double mutants are a result of NHEJ repair of meiotic DSBs, we depleted *lig-4* in *smc-5(ok2421); him-6(ok412)* double mutants. In *smc-5(ok2421); lig-4; him-6(ok412)* triple mutants chromatin bridges still occurred (S6 Fig), indicating that ligase IV does not contribute for the formation of chromatin bridges in *smc-5(ok2421); him-6(ok412)* double mutants.

### Loss of BRC-1/BRD-1 rescues the progeny lethality of *smc-5; him-6* mutants and suppresses chromatin bridge formation

**Fig 5:**
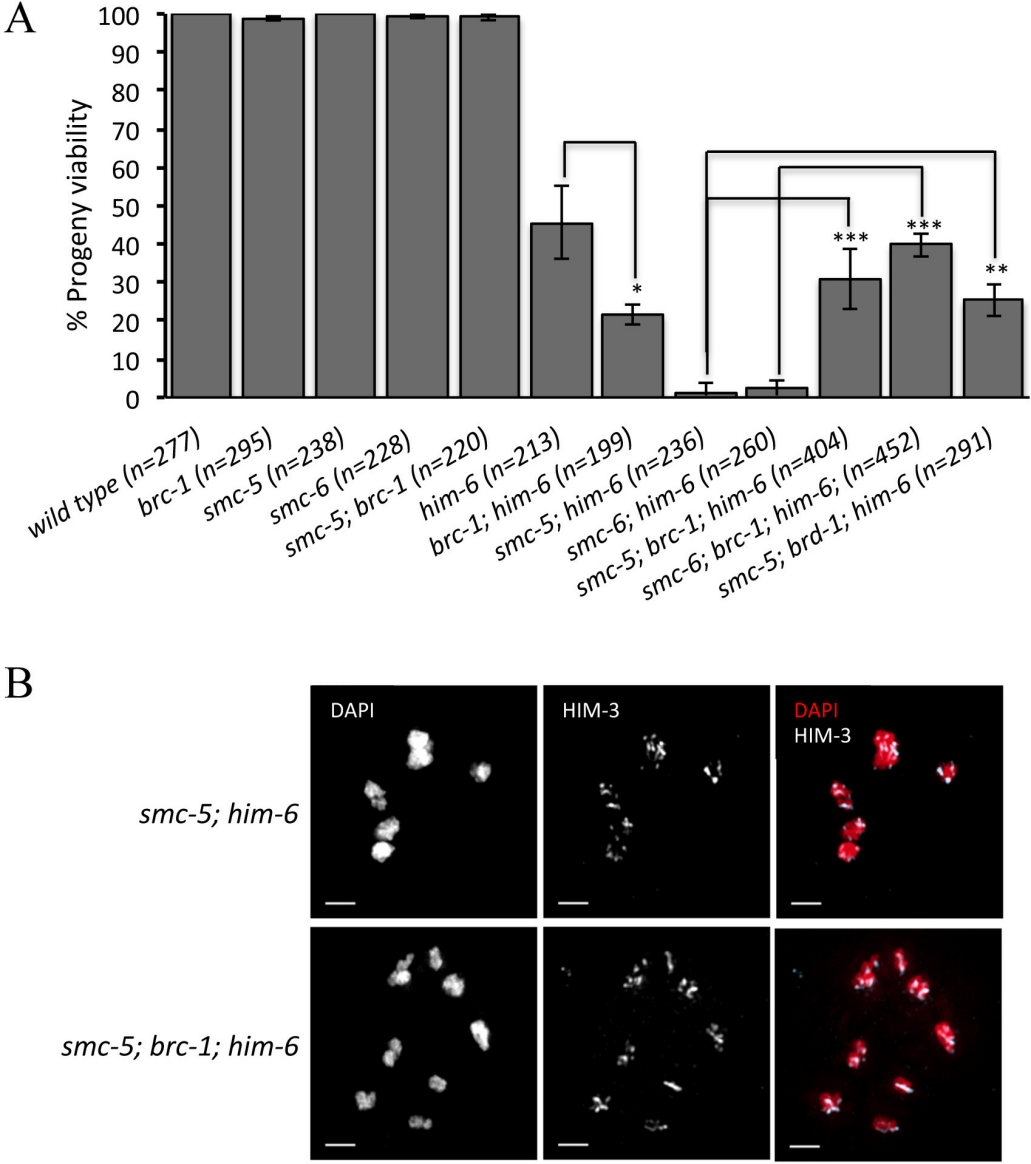
Loss of BRC-1/BRD-1 rescues the progeny lethality of *smc-5; him-6* double mutants and suppresses the chromatin bridge formation. **A.** Mutation of *brc-1* or *brd-1* rescues the lethality of *smc-5(ok2421); him-6(ok412)* double mutants. The progeny viability (%) data are represented as averages of three independent experiments and error bars represent standard deviation. The sample size (n) indicates the number of embryos examined for each genotype. Asterisks indicate statistical significance as determined by two-tailed Student T-test. P Values below 0.05 were consider significant, where p < 0.05 was indicated with *, p < 0.01 with ** and p < 0.005 with ***. **B.** Representative images of chromosomes in -1 oocytes at diakinesis stained with DAPI and an antibody recognizing the chromosome axis component HIM-3. Scale bars: 2 μm.

A recent study reported that *smc-5* worms show reduced survival in response to perturbed DNA replication and that this increased sensitivity could be alleviated by *brc-1* mutations [66]. The rescue of *smc-5/6* mutant defects by loss of *brc-1 or brd-1* (the *Bard1* ortholog) was likely due to the suppression of HR activity. To test whether the BRC-1/BRD-1 mutation could also suppress the meiotic defect of *smc-5(ok2421); him-6(ok412)* mutants, we depleted BRC-1/BRD-1 in *smc-5(ok2421); him-6(ok412)* mutants. As previously reported, worms lacking BRC-1 are viable (Fig 5A) [10]. Combining *brc-1* and *smc-5* did not result in a reduction in progeny viability (Fig 5A), while viability dropped from 45% in *him-6(ok412)* to 21%, in *brc-1; him-6(ok412)* double mutants (Fig 5A, p<0.05). Importantly, we found that deletion of BRC-1/BRD-1 complex partially rescued the lethality of *smc-5(ok2421); him-6(ok412)* and *smc-6(ok3297); him-6(ok412)* mutants to a level of 25%-40% (Fig 5A). Cytological analysis revealed that *smc-5(ok2421); brc-1; him-6(ok412)* triple mutants had no chromatin bridges and 20% of the oocytes we checked possessed univalents as manifested by more than six DAPI-stained bodies (Fig 5B and S5 Fig), suggesting that the toxic chromatin bridges in *smc-5(ok2421); him-6(ok412)* mutants were BRC-1/BRD-1-dependent.

**Fig 6:**
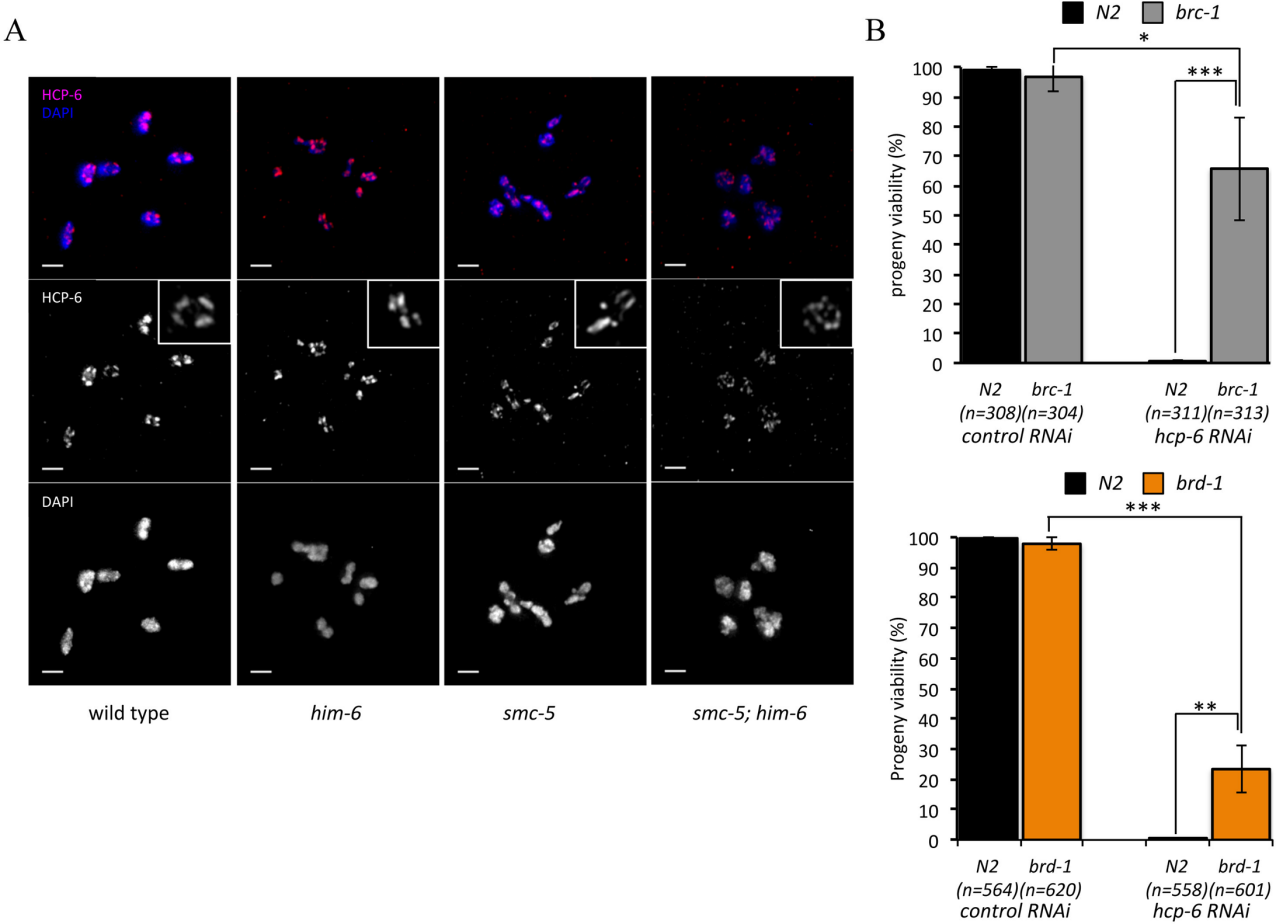
SMC-5/6 complex and HIM-6 are important for controlling chromosome structure during meiosis. **A.** Analysis of distribution of condensin on chromosomes of wild type and various indicated mutants. The condensin II complex was visualized with HCP-6 antibody. Scale bars: 2 μm. **B.** Mutation of *brc-1* partially rescued the progeny lethality caused by *hcp-6* RNAi. The progeny viability (%) data are represented as averages of three independent experiments and error bars represent standard deviation. The sample size (n) indicates the total number of embryos examined for each genotype. Asterisks indicate statistical significance as determined by two-tailed Student T-test. P Values below 0.05 were consider significant, where p < 0.05 was indicated with *, p < 0.01 with ** and p < 0.005 with ***.

Chromosomes undergo extensive structural reorganization to achieve accurate chromosome segregation during meiosis [67]. Chromosome condensation involves the remodeling of each pair of homologous chromosomes around the site of CO into highly condensed cruciform bivalents [68]. Mutation of *hcp-6*, a component of condensin II, leads to a dramatically decreased number of cruciform bivalents and defective chromosome segregation during meiotic cell divisions [69, 70]. The phenotypes reported in *hcp-6* mutants are reminiscent of the phenotypes we observed in in *smc-5(ok2421); him-6(ok412)* double mutants [70], indicating that the depletion of SMC-5/6 and HIM-6 could similarly lead to a defect in higher-ordered chromosome organization. We further examined the distribution of condensin in wild type and the various single and double mutants by HCP-6 staining. In the wild type and the *him-6(ok412)* mutant, HCP-6 is associated with sister chromatids and formed four distinct well organized patches in each bivalent (Fig 6A). In the *smc-5(ok2421)* single mutant, although HCP-6 still formed patches, an increased number of patches were detected in some bivalents (Fig 6A). However, in the in *smc-5(ok2421); him-6(ok412)* double mutant HCP-6 staining is scattered along the bivalent and no distinct well-organized patches can be observed, indicating that the distribution of condensin is severely compromised in the absence of SMC-5/6 and HIM-6 (Fig 6A). *hcp-6* RNAi has previously been shown to lead to a partial HCP-6 depletion in the germ line and, to the formation of chromatin bridges during meiotic divisions [70]. Given that the embryonic lethality of in *smc-5(ok2421); him-6(ok412)* can be partially rescued by depletion of BRC-1/BRD-1 and that the chromatin bridges in in *smc-5(ok2421); him-6(ok412)* mutant are *brc-1* dependent, we tested whether mutation of *brc-1 or brd-1* could also rescue the lethality conferred by *hcp-6* depletion. As expected we found that the RNAi depletion of HCP-6 in wild type cause 100% embryonic lethality. However progeny viability is rescued to 65% and 23% when HCP-6 is depleted in *brc-1* and *brd-1* respectively (Fig 6B), suggesting that BRC-1/BRD-1 may be also required for the formation of toxic chromatin bridges when chromosome condensation is defective during meiosis.

## Discussion

In this study we examined the synergistic functions of the BLM helicase and SMC-5/6 during *C*. *elegans* meiosis. We show that BLM helicase and SMC-5/6 complex cooperate to process meiotic recombination intermediates and promote the maturation into structured bivalents. We also uncover an unexpected role of HIM-6 in regulating CO distribution.

I) HIM-6 and SMC-5/6 are required for normal CO formation and distribution

**Fig 7:**
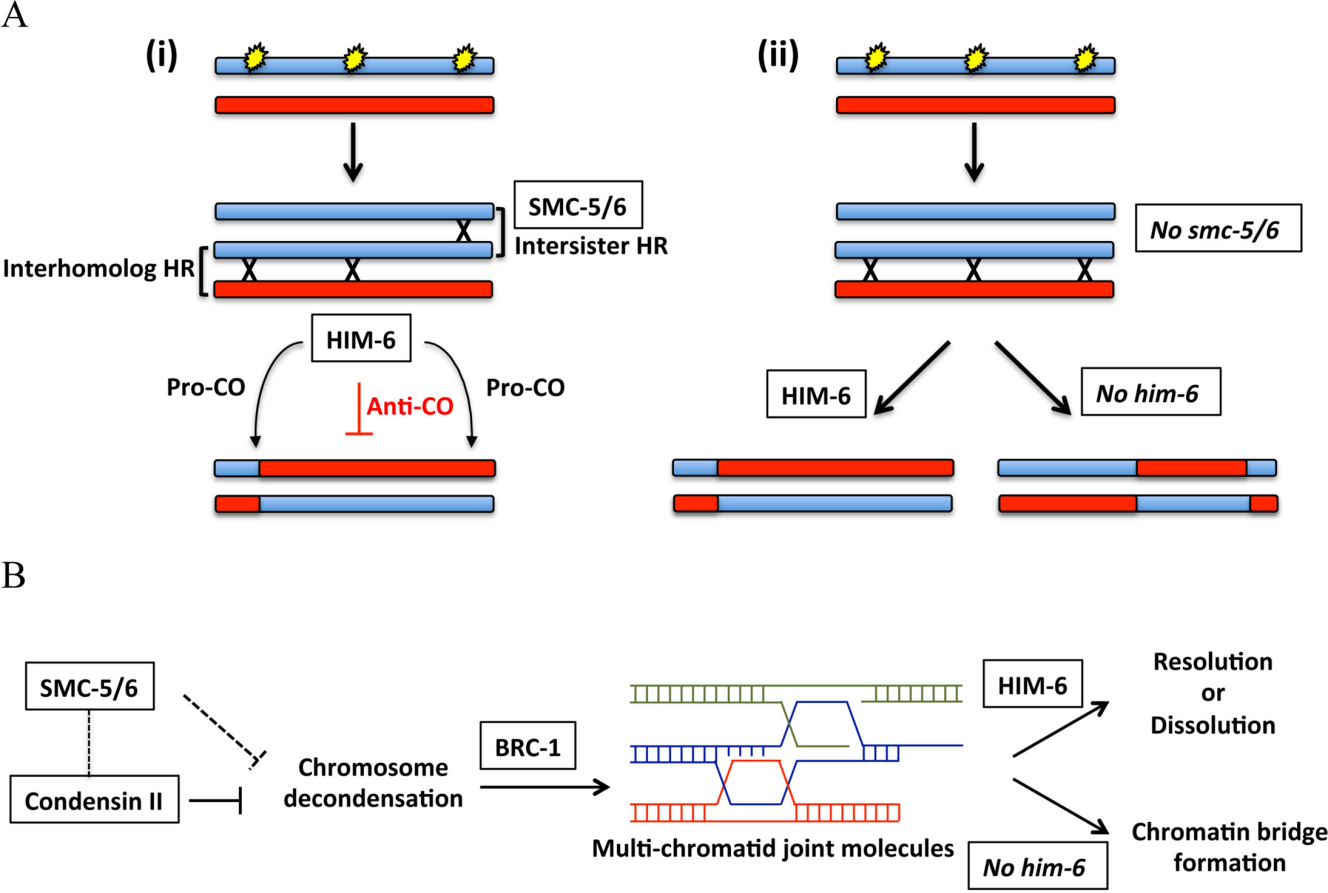
Models of the cooperation between SMC-5/6 complex and HIM-6 in meiotic recombination intermediate metabolism. **A.** Schematic diagram depicting the CO formation in the wild type (i) or in the *smc-5/6* mutants (ii). In the wild type, SMC-5/6 complex can promote the repair of SPO-11 induced DSBs by intersister recombination pathways. The CO formation generated by interhomolog recombination can be regulated by HIM-6. However, in the absence of SMC-5/6, more DSBs are channelled to be repaired by interhomolog recombination due to the compromised intersister recombination. In this case, HIM-6 becomes essential to maintain a normal CO landscape. **B.** Reduced chromosome compaction caused by *smc-5/6* and *him-6* mutation or by condensin depletion may lead to formation of multi-chromatid joint molecules supressed by *brc-1*. The dashed lines represent a potential role of SMC-5/6 in preventing the chromosome decondensation probably through cross-talk between SMC-5/6 complex and condensin II complex.

In all organisms studied so far, a significant excess of DSBs are generated relative to the number of CO [49, 71–75]. In *C*. *elegans*, although each chromosome undergoes several DSBs, only one DSB results in interhomolog CO between each chromosome pair. The remainder are repaired either by interhomolog non-CO or the intersister recombination pathway [76]. BLM helicase is highly conserved, but the functions of BLM in CO formation differ in various organisms. The budding yeast BLM homologue Sgs1 shows anti-CO activity. Mutation of *sgs1* led to 50% increase in CO frequency [77]. In contrast, the fission yeast BLM homologue Rqh1 is able to promote the recombination outcome towards CO formation [78]. Similarly, meiotic recombination is reduced to about half of the wild type frequency in *D*. *melanogaster* in the absence of the BLM ortholog mus309 [79]. In *C*. *elegans*, the BLM homolog HIM-6 has been found to be required for normal levels of recombination during meiosis [16, 41, 54, 55]. Mutations in *him-6* lead to a decrease in progeny viability, reduced number of COs between homolog chromosomes and increased univalent formation. Interestingly, our careful examination of CO frequency and distribution revealed that although the overall CO level is decreased in *him-6* mutants, *him-6* leads to a strong reduction of COs in the arm regions while the frequency of COs in the central region is significantly increased. Our findings, which are based on the analysis of two chromosomes, are consistent with a previous study showing an altered CO frequency and distribution in *him-6* mutants [55]. Our data are also supported by a recent publication showing the same effect when *rmh-1* encoding for a regulatory subunit of HIM-6/Blooms is deleted [56]. Thus, HIM-6 might have a pro-CO activity at arm regions but have an anti-CO activity in the center of chromosomes (Fig 7A). How is CO distribution regulated by HIM-6 in *C*. *elegans* meiosis? In mice, the BLM helicase colocalizes with the recombinases RAD51 and DMC1 and probably has an early role in the CO/non-CO decision [80]. Additionally, the BLM helicase has been shown to interact physically or functionally with different partners [81]. On one hand, BLM can form a complex with TopIIIα and RMI and is able to dissolve double HJ to generate non-CO [82]. On the other hand, the *C*. *elegans* BLM helicase HIM-6 can also act with XPF-1 endonuclease as a HJ resolvase to promote CO formation [16]. Therefore, the pro- or anti-CO activity of BLM on different regions of chromosome might be conferred by different BLM complexes. Notably, the CO distribution shifting from the arm region to the center of chromosome has also been reported for the *C*. *elegans rec-1*, *xnd-1* and *him-5* mutants and a weaker such phenotype also occurs in *slx-1* [53, 83–85]. While XND-1 is thought to regulate CO distribution by modulating acetylation levels of histone H2A lysine 5, REC-1 and HIM-5 act redundantly to facilitate the formation of meiotic DSBs and the phosphorylation of REC-1 by cyclin-dependent kinase could be important for the CO distribution [86].

At the moment we can only speculate as to why recombination is increased at the center of the chromosomes in *him-6* mutants, why this effect is stronger in *smc-5(ok2421); him-6(ok412)* double mutants, and why CO recombination is generally increased in this double mutant. Notably, neither *smc-5(ok2421); him-6(ok412)* double mutants nor the corresponding single mutants show an excessive number of COSA-1 foci, arguing that ‘excessive recombination’ in the double mutants might not be due to an increased number of CO designated sites, but might reflect COs outcome of ectopic recombination intermediates. Such increased recombination could be due to delayed disassembly of the synaptonemal complex observed in *smc-5(ok2421); him-6(ok412)* double mutants, as indicated by a prolonged SYP-1 staining on the bivalents in the -1 oocytes at diakinesis. Such delayed desynapsis could promote interhomolog recombination to form excessive COs and could also account for the increased chance for COs occurring at the central chromosomal regions. However, this model cannot account for increased recombination at the center of *him-6* chromosomes as we did not observe a desynapsis delay in *him-6* single mutants. Furthermore, also not consistent with this model, the frequency and distribution of COs are not altered in *smc-5* single mutants, despite desynapsis being delayed.

In budding yeast, the Smc5/6 complex is critical for interhomolog bias by promoting the orderly formation of interhomolog recombination intermediates [32–34]. In contrast, we confirmed a previous study showing that the SMC-5/6 complex is not required for CO formation in *C*. *elegans* and has a weak or negligible effect on interhomolog recombination [43]. This previous study also revealed that when interhomolog recombination is disrupted by depletion of the chromosome axis component HIM-3 or the synaptonemal complex element SYP-2 in *smc-5/*6 mutants, chromosome fragments occur. This phenotype indicates an important function of SMC-5/6 in promoting intersister recombination during meiosis in *C*. *elegans* [43]. Therefore, in line with previous studies and our genetic and recombination mapping data, we propose that SMC-5/6 and HIM-6 act synergistically to repair meiotic DSBs and regulate CO formation (Fig 7A). While HIM-6 is required for interhomolog recombination, a proportion of meiotic DSBs are repaired by the SMC-5/6 promoted intersister recombination. Compromised intersister recombination repair caused by loss of SMC-5/6 complex probably results in more DSBs to be repaired by interhomolog recombination. In such a scenario increased interhomolog recombination might be quelled by HIM-6, the additional depletion of which would allow for excessive CO formation in addition to an abnormal CO interference.

II) SMC-5/6 complex and HIM-6 are required for correct chromosome organization during meiosis

The SMC complexes, cohesion, condensin and SMC-5/6 complex, are essential for chromosome organization and dynamics. Numerous studies have revealed that chromosome morphogenesis is important for meiotic recombination regulation and CO formation [67]. Condensin is essential for preventing/resolving chromatin bridges formation probably by maintaining an organized and ordered chromosome structure during meiotic recombination [70]. Depletion of Smc5/6 in human mitotic cells led to a decondensed chromosome conformation accompanied by defective condensin localization, indicating that there might be cross-talk between Smc5/6 and condensin [87]. Related to this, we show that the chromosome organization and condensin distribution are abnormal in *smc-5(ok2421)* and *smc-6(ok3297)* mutants in *C*. *elegans*. Further depletion of the BLM helicase HIM-6 in *smc-5(ok2421)* mutant led to much more severe defects in chromosome organization. The phenotype we observe is reminiscent to condensation defects, a disrupted chiasma structure and the formation of chromatin bridges that occurs upon HCP-6 depletion [69, 70]. The partial decondensation of pachytene chromosomes in dosage compensation mutants (for instance *dpy-28*) has been previously linked to increased recombination [88, 89]. Thus the excessive recombination occurring in *smc-5(ok2421); him-6(ok412)* could be due to the failure to form properly condensed chromosomes. While we have not observed overt chromosome decondensation in *smc-5(ok2421); him-6(ok412)* mutants during pachytene, the exact state of those pachytene chromosomes remains to be investigated.

How could *him-6* mutations enhance the chromosome disorganization observed in *smc-5/6* mutants? It is possible that defective chromosome organization in *smc-5(ok2421); him-6(ok412)* double mutants could be partially due to the compromised meiotic DSBs repaired by HR. An extreme case of such a situation occurs in *rad-51* mutants, where *spo-11* dependent DSBs cannot be repaired by HR, leading to an uncondensed mass of chromatin [90]. It is also possible that the frequency of ectopic recombination could be increased when the chromosome is decondensed (Fig 7B). Toxic recombination intermediates generated by ectopic recombination, such as multi-chromatid joint molecules in budding yeast, are resolved or dissolved by BLM helicase homolog Sgs1 [91, 92]. In the absence of BLM helicase, unresolved multi-chromatid intermediates could lead to the formation of aberrant chromosome organization and chromatin bridges. Interestingly, we find that depleting the BRCA1 ortholog BRC-1 could rescue the progeny lethality conferred by *smc-5(ok2421); him-6(ok412)* mutants and by the *RNAi* depletion of *hcp-6*. BRCA1 plays a role in meiotic DNA-damage repair and CO formation during spermatogenesis in mice [39]. BRCA1 deficiency resulted in decreased number of MSH4 foci and delayed appearance of MLH1 foci, indicating that mouse BRCA1 could be involved in regulating efficient formation or stabilization of meiotic recombination intermediates [38]. Consistent with this possibility, we found that depletion of BRC-1 in *smc-5(ok2421); him-6(ok412)* double mutants and *hcp-*6 *(RNAi)* worms suppressed chromatin bridge formation in *C*. *elegans* during meiosis, suggesting that BRC-1 might promote ectopic recombination by driving the formation or stabilization of aberrant joint molecules between non homologous chromosomes and between chromatids, possibly leading to multi-chromatid linkage formation (Fig 7B).

In conclusion, our results suggest that the BLM helicase HIM-6 and the SMC-5/6 complex act synergistically to promote recombination intermediate processing and chromosome maturation during *C*. *elegans* meiosis. We also reveal a role of the BLM helicase HIM-6 in regulating CO distribution. Finally, we highlight an important role of chromosome architecture in preventing ectopic meiotic recombination.

## Materials and Methods

### *C*. *elegans* strains and maintenance

All strains were maintained at 20°C under standard conditions. N2 Bristol was used as the wild type strain. CB4856 Hawaii was used to generate strains for CO recombination frequency analysis. Mutant strains used in this study are listed in S1 Table.

All the mutants used in this study were obtained from the *Caenorhabditis* Genetics Center. Details are described at the National Bioresource Project for the nematode and on www.wormbase.org. All mutants were outcrossed for a minimum of four times to the wild type strain to eliminate background mutations. The TG2512 *gtIs2512 [Ppie-1*::*his-11*::*GFP unc-119+]* strain was generating by biolistic bombardment of pAZ132 of *unc-119(ed3)* mutants.

### Cytological procedures

For immunostaining of germlines, 8 to 10 (24 h post L4 stage) adults were dissected per slide. Germlines were isolated in 8 μl of 1×dissection buffer (250 mM HEPES pH 7.4, 1.18 M NaCl, 480 mM KCl, 20 mM EDTA, 5 mM EGTA, 0.1% Tween 20, 20 mM sodium azide). An equal volume of 2% formaldehyde was added to the slide carefully pipetting to allow for homogenization. Fixation was done for 5 minutes at room temperature, followed by immersion in liquid nitrogen. Coverslips were quickly removed, and post fixation was done in −20°C methanol/acetone (50%/ 50%) for 10 minutes, followed by permeabilization by washing 3×10 minutes in PBST (1×PBS, 0.1% Tween) at room temperature. Blocking was performed in PBST supplemented with 1% BSA (PBSTB) incubated for 30 min at room temperature. Primary antibodies were diluted in PBSTB and covered with a parafilm coverslip, followed by over-night incubation at 4°C in a dark humid chamber. Slides were then washed 3×10 min in PBST. Secondary antibody incubation was done at room temperature for 2 hours in PBSTB supplemented with 2 μg/μL DAPI. After washing 3×10 min in PBST and 5 min in PBS the samples were mounted in Vectashield mounting medium (Vector Laboratories, Inc.) and sealed. Primary and secondary antibodies were used at the indicated dilutions: rabbit anti-HTP-3 (1:500) and anti-HCP-6 (1:250) [70]; guinea pig anti-SYP-1 (1:500), and Alexa 568 labelled donkey anti-rabbit (1:750) (Molecular Probes).

### RNA interference

RNA interference by feeding was performed using bacteria RNAi feeding strains from the Ahringer library [93]. L4 worms were placed on plates seeded with bacteria expressioning dsRNA. After 24 hours, the adult animals were transferred to new RNAi plates and allowed to lay eggs for approximately 6 hours. The resulting progeny viability for wild type, *brc-1* and *brd-1* mutants were scored. Assays were performed at 20°C. Bacteria containing empty RNAi vector L4440 or mcm-7 RNAi vector were used for control experiments.

### Recordings of meiotic divisions

Embryos were dissected in isotonic growth medium for blastomeres containing 35% bovine FCS. Before use, bovine FCS (heat treated for 30 min at 56°C; Invitrogen) was added. Embryos were mounted on 2% agarose pads. Vaseline patches on the slide were used to reduce the pressure of the coverslip on the embryo. Images were captured every 10 seconds using a widefield DeltaVision microscope. Exposure time was 250 milliseconds and binning used was 2×2. Image analysis and video processing were performed using ImageJ software,

### Image acquisition

Microscopy images were acquired with a Delta Vision Image restoration system (Applied Precision). Raw data obtained were analysed and deconvolved using SoftWoRx Suite and softWoRx Explorer software (Applied Precision, Issaquah, WA, USA). For SIM microscopy, established protocols were followed [16]. Images were acquired using a UPlanSApochromat 100× 1.4NA, oil immersion objective lens (Olympus, Center Valley, PA) and back-illuminated Cascade II 512×512 electron-multiplying charge-coupled device (EMCCD) camera (Photometrics, Tucson, AZ) on the SIM version 3 system (Applied Precision) equipped with 405-, 488-, and 593-nm solid-state lasers. Samples were illuminated by a coherent scrambled laser light source that had passed through a diffraction grating to generate the structured illumination by interference of light orders in the image plane to create a 3D sinusoidal pattern, with lateral stripes approximately 0.2 μm apart. The pattern was shifted laterally through five phases and through three angular rotations of 60° for each Z-section, separated by 0.125 μm. Exposure times were typically between 100 and 200 ms, and the power of each laser was adjusted to achieve optimal intensities of between 2,000 and 4,000 counts in a raw image of 16-bit dynamic range, at the lowest possible laser power to minimize photo bleaching. Raw images were processed and reconstructed to reveal structures with greater resolution implemented on SoftWoRx, ver. 6.0 (Applied Precision, Inc.). The channels were then aligned in x, y, and rotationally using predetermined shifts as measured using 100 nm TetraSpeck (Invitrogen) beads with the SoftWoRx alignment tool (Applied Precision, Inc.).

### Determining meiotic crossover recombination frequencies

Meiotic CO recombination frequencies were assayed essentially as described, using five snip-SNPs on Chromosome V that differ between N2 Bristol and CB4856 Hawaii [51]. Strains used to determine CO recombination assays were crossed into Hawaii to obtain mutant strains carrying ChrV homozygous for Hawaii DNA. Single and double mutant strains containing *smc-5* were balanced with mIn1. GFP positive balanced mutant males with ChrV homozygous Hawaii were then crossed with hermaphrodites of identical genotype in the N2 Bristol background to obtain mutant strains heterozygous for Hawaii. Non-GFP homozygous mutant F1 cross-progeny hermaphrodites were then crossed with males of CB5584, a *myo-2*::GFP expressing strain, which expresses high levels of green fluorescent protein in pharyngeal muscles, allowed to lay eggs for 24–48 h before removing them for genotype confirmation by PCR and *Dra*I digest. 100–200 individual F1′ GFP-positive embryos and larvae were lysed and analysed for CO recombination by PCR and *Dra*I digest.

Primers used:

Chromosome I

−19:    3’- ATGCCAGTGATAAGGAACGG-5’

           3’- TCACATCCCTTGTCGATGAA-5’

−6:      3’- GTTTTCACTTTTGCCGGTGT-5’

           3’- TGAAGGCGCATATACAGCAG-5’

5:        3’- ATCTGGCACCAAATATGAGTCG -5’

           3’- AATCTCGATTTTCAAGGAGTGG -5’

14:      3’- TTGAAATCCCCTTTAAAATCCC -5’

           3’- ACACTGGGTACCTGACTCATGC -5’

26:       3’- ATTATTAACGGCCACGGTGA -5’

           3’- CCCACACACTCTCACCTTCA -5’

Chromosome V

−17.5: 3’-TTTCGGAAAATTGCGACTGT-5’

           3’-CGCGTTTTGGAGAATTGTTT-5’

−5:      3’-GAGATTCTAGAGAAATGGACACCC-5’

           3’-AAAAATCGACTACACCACTTTTAGC-5’

5.8:     3’-CAAATTAAATATTTCTCAAAGTTTCGG-5’

           3’-ACATAAGCGCCATAACAAGTCG-5’

17.8:   3’-GAAATTCAAATTTTTGAGAAACCC-5’

           3’-TTCAGACCATTTTTAGAATATTCAGG-5’

25.1:   3’-ACTTGACTCCTCTTTTCCATG-5’

           3’-CTGCTAGCTCAAATACTCCC-5’

## Supporting Information

S1 Fig Representative images of germline nuclei from wild type, *him-6(ok412)*, *smc-5(ok2421) and smc-5(ok2421); him-6(ok412)* stained with DAPI (blue) and anti-RAD-51 antibody (red). (TIF)

S2 Fig Meiotic chromosome axis formation and synapsis are normal in *smc-5(ok2421); him-6(ok412)* double mutants. **A.** Representative images of pachytene nuclei stained with an antibody recognizing the chromosome axis component HTP-3 (green) and DAPI (blue). Scale bars: 1 μm. **B.** SYP-1 immunostaining of representative pachytene nuclei. Scale bars: 2 μm.
(TIF)

S3 Fig Analysis of crossover frequencies and distribution on chromosome I of wild type and *him-6* mutants. The genetic map positions of the five SNPs, which together cover 87% of chromosome I, are indicated. n is the number of cross-progeny scored. The frequency of 2 COs, 1 CO or 0 CO per chromosome is indicated in absolute numbers and as percentage (in brackets). The relative recombination frequencies (mutant/ wild type) are indicated by different coloured tags. Red reflects the greatest increase and green reflects the greatest decrease.
(TIF)

S4 Fig Representative images of nuclei of diakinesis oocytes stained with an antibody recognizing the chromosome axis component HIM-3. (TIF)

S5 Fig Quantification of the number of DAPI-stained bodies in -1 diakinesis oocytes of wild type, *smc-5(ok2421)*, *him-6(ok412)*, *smc-5(ok2421); him-6(ok412)* and *smc-5(ok2421); brc-1; him-6(ok412)* mutants. The sample size (n) indicates the total number of -1 diakinesis oocytes examined for each genotype. The proportion of oocytes with six DAPI staining body in *him-6* (11.8%, n = 51), *smc-5; him-6* double mutants (46.7%, n = 45) and *smc-5; brc-1; him-6* (80.6%, n = 31) were significantly different from the wild type (100%, n = 49) (p<0.05). There is no difference between wild type (100%, n = 49) and *smc-5* (97.4%, n = 39) (p = 0.258). Statistical significance was determined by two-tailed Z-test for two population proportions. P Values below 0.05 were considered significant.
(TIF)

S6 Fig Representative images of DAPI-stained diakinesis chromosomes of *smc-5(ok2421); lig-4; him-6(ok412)* triple mutants. Scale bars: 2 μm.
(TIF)

S1 Table List of strains used in this study. (DOCX)

S1 Video Wild-type histone::GFP, meiosis I and II. (AVI)

S2 Video *him-6(ok412);* histone::GFP, meiosis I and II. (AVI)

S3 Video *smc-5(ok2421);* histone::GFP, meiosis I and II. (AVI)

S4 Video *smc-5(ok2421); him-6(ok412);* histone::GFP, meiosis I and II. (AVI)

S5 Video DAPI and anti-HIM-3 antibody stained diakinesis chromosomes of *smc-5; him-6(ok412)*. (MOV)

## References

[1] Mimitou EP, Symington LS (2009) Nucleases and helicases take center stage in homologous recombination. Trends Biochem Sci 34: 264–272. 10.1016/j.tibs.2009.01.010 19375328

[2] Baudat F, Imai Y, de Massy B (2013) Meiotic recombination in mammals: localization and regulation. Nat Rev Genet 14: 794–806. 10.1038/nrg3573 24136506

[3] Keeney S, Giroux CN, Kleckner N (1997) Meiosis-specific DNA double-strand breaks are catalyzed by Spo11, a member of a widely conserved protein family. Cell 88: 375–384. 9039264 10.1016/s0092-8674(00)81876-0

[4] Humphryes N, Hochwagen A (2014) A non-sister act: Recombination template choice during meiosis. Exp Cell Res 329: 53–60. 10.1016/j.yexcr.2014.08.024 25158281 PMC4561180

[5] Phadnis N, Hyppa RW, Smith GR (2011) New and old ways to control meiotic recombination. Trends Genet 27: 411–421. 10.1016/j.tig.2011.06.007 21782271 PMC3177014

[6] Pradillo M, Santos JL (2011) The template choice decision in meiosis: is the sister important? Chromosoma 120: 447–454. 10.1007/s00412-011-0336-7 21826413

[7] Goldfarb T, Lichten M (2010) Frequent and efficient use of the sister chromatid for DNA double-strand break repair during budding yeast meiosis. PLoS Biol 8: e1000520 10.1371/journal.pbio.1000520 20976044 PMC2957403

[8] Rosu S, Libuda DE, Villeneuve AM (2011) Robust crossover assurance and regulated interhomolog access maintain meiotic crossover number. Science 334: 1286–1289. 10.1126/science.1212424 22144627 PMC3360972

[9] Couteau F, Zetka M (2011) DNA damage during meiosis induces chromatin remodeling and synaptonemal complex disassembly. Dev cell 20: 353–363. 10.1016/j.devcel.2011.01.015 21397846

[10] Adamo A, Montemauri P, Silva N, Ward JD, Boulton SJ, et al (2008) BRC-1 acts in the inter-sister pathway of meiotic double-strand break repair. EMBO Rep 9: 287–292. 10.1038/sj.embor.7401167 18219312 PMC2267377

[11] Bellendir SP, Sekelsky J (2013) An elegans Solution for Crossover Formation. PLoS Genet 9: e1003658 10.1371/journal.pgen.1003658 23874241 PMC3715449

[12] Zakharyevich K, Tang S, Ma Y, Hunter N (2012) Delineation of joint molecule resolution pathways in meiosis identifies a crossover-specific resolvase. Cell 149: 334–347. 10.1016/j.cell.2012.03.023 22500800 PMC3377385

[13] Matos J, Blanco MG, Maslen S, Skehel JM, West SC (2011) Regulatory control of the resolution of DNA recombination intermediates during meiosis and mitosis. Cell 147: 158–172. 10.1016/j.cell.2011.08.032 21962513 PMC3560330

[14] Ip SC, Rass U, Blanco MG, Flynn HR, Skehel JM, et al (2008) Identification of Holliday junction resolvases from humans and yeast. Nature 456: 357–361. 10.1038/nature07470 19020614

[15] De Muyt A, Jessop L, Kolar E, Sourirajan A, Chen J, et al (2012) BLM helicase ortholog Sgs1 is a central regulator of meiotic recombination intermediate metabolism. Mol Cell 46: 43–53. 10.1016/j.molcel.2012.02.020 22500736 PMC3328772

[16] Agostinho A, Meier B, Sonneville R, Jagut M, Woglar A, et al (2013) Combinatorial regulation of meiotic holliday junction resolution in C. elegans by HIM-6 (BLM) helicase, SLX-4, and the SLX-1, MUS-81 and XPF-1 nucleases. PLoS Genet 9: e1003591 10.1371/journal.pgen.1003591 23901331 PMC3715425

[17] O'Neil NJ, Martin JS, Youds JL, Ward JD, Petalcorin MI, et al (2013) Joint molecule resolution requires the redundant activities of MUS-81 and XPF-1 during Caenorhabditis elegans meiosis. PLoS Genet 9: e1003582 10.1371/journal.pgen.1003582 23874209 PMC3715453

[18] Saito TT, Lui DY, Kim HM, Meyer K, Colaiacovo MP (2013) Interplay between structure-specific endonucleases for crossover control during Caenorhabditis elegans meiosis. PLoS Genet 9: e1003586 10.1371/journal.pgen.1003586 23874210 PMC3715419

[19] De Piccoli G, Torres-Rosell J, Aragon L (2009) The unnamed complex: what do we know about Smc5-Smc6? Chromosome Res 17: 251–263. 10.1007/s10577-008-9016-8 19308705

[20] Cobbe N, Heck MM (2004) The evolution of SMC proteins: phylogenetic analysis and structural implications. Mol Biol Evol 21: 332–347. 14660695 10.1093/molbev/msh023

[21] Jeppsson K, Kanno T, Shirahige K, Sjogren C (2014) The maintenance of chromosome structure: positioning and functioning of SMC complexes. Nat Rev Mol Cell Biol 15: 601–614. 10.1038/nrm3857 25145851

[22] Losada A, Hirano T (2005) Dynamic molecular linkers of the genome: the first decade of SMC proteins. Genes Dev 19: 1269–1287. 15937217 10.1101/gad.1320505

[23] Fujioka Y, Kimata Y, Nomaguchi K, Watanabe K, Kohno K (2002) Identification of a novel non-structural maintenance of chromosomes (SMC) component of the SMC5-SMC6 complex involved in DNA repair. J Biol Chem 277: 21585–21591. 11927594 10.1074/jbc.M201523200

[24] Kegel A, Sjogren C (2010) The Smc5/6 complex: more than repair? Cold Spring Harb Symp Quant Biol 75: 179–187. 10.1101/sqb.2010.75.047 21467147

[25] Andrews EA, Palecek J, Sergeant J, Taylor E, Lehmann AR, et al (2005) Nse2, a component of the Smc5-6 complex, is a SUMO ligase required for the response to DNA damage. Mol Cell Biol 25: 185–196. 15601841 10.1128/MCB.25.1.185-196.2005 PMC538766

[26] Potts PR, Yu H (2005) Human MMS21/NSE2 is a SUMO ligase required for DNA repair. Mol Cell Biol 25: 7021–7032. 16055714 10.1128/MCB.25.16.7021-7032.2005 PMC1190242

[27] Onoda F, Takeda M, Seki M, Maeda D, Tajima J, et al (2004) SMC6 is required for MMS-induced interchromosomal and sister chromatid recombinations in Saccharomyces cerevisiae. DNA repair 3: 429–439. 15010319 10.1016/j.dnarep.2003.12.007

[28] Murray JM, Carr AM (2008) Smc5/6: a link between DNA repair and unidirectional replication? Nat Rev Mol Cell Biol 9: 177–182. 18059412 10.1038/nrm2309

[29] Gomez R, Jordan PW, Viera A, Alsheimer M, Fukuda T, et al (2013) Dynamic localization of SMC5/6 complex proteins during mammalian meiosis and mitosis suggests functions in distinct chromosome processes. J Cell Sci 126: 4239–4252. 10.1242/jcs.130195 23843628 PMC3772391

[30] Verver DE, van Pelt AM, Repping S, Hamer G (2013) Role for rodent Smc6 in pericentromeric heterochromatin domains during spermatogonial differentiation and meiosis. Cell Death Dis 4: e749 10.1038/cddis.2013.269 23907463 PMC3763431

[31] Verver DE, Langedijk NS, Jordan PW, Repping S, Hamer G (2014) The SMC5/6 complex is involved in crucial processes during human spermatogenesis. Biol Reprod 91: 22 10.1095/biolreprod.114.118596 24855106 PMC6058740

[32] Copsey A, Tang S, Jordan PW, Blitzblau HG, Newcombe S, et al (2013) Smc5/6 coordinates formation and resolution of joint molecules with chromosome morphology to ensure meiotic divisions. PLoS Genet 9: e1004071 10.1371/journal.pgen.1004071 24385939 PMC3873251

[33] Lilienthal I, Kanno T, Sjogren C (2013) Inhibition of the Smc5/6 complex during meiosis perturbs joint molecule formation and resolution without significantly changing crossover or non-crossover levels. PLoS Genet 9: e1003898 10.1371/journal.pgen.1003898 24244180 PMC3820751

[34] Xaver M, Huang L, Chen D, Klein F (2013) Smc5/6-Mms21 prevents and eliminates inappropriate recombination intermediates in meiosis. PLoS Genet 9: e1004067 10.1371/journal.pgen.1004067 24385936 PMC3873250

[35] Wehrkamp-Richter S, Hyppa RW, Prudden J, Smith GR, Boddy MN (2012) Meiotic DNA joint molecule resolution depends on Nse5-Nse6 of the Smc5-Smc6 holocomplex. Nucleic Acids Res 40: 9633–9646. 10.1093/nar/gks713 22855558 PMC3479181

[36] Huen MS, Sy SM, Chen J (2010) BRCA1 and its toolbox for the maintenance of genome integrity. Nat Rev Mol Cell Biol 11: 138–148. 10.1038/nrm2831 20029420 PMC3899800

[37] Scully R, Chen J, Plug A, Xiao Y, Weaver D, et al (1997) Association of BRCA1 with Rad51 in mitotic and meiotic cells. Cell 88: 265–275. 9008167 10.1016/s0092-8674(00)81847-4

[38] Broering TJ, Alavattam KG, Sadreyev RI, Ichijima Y, Kato Y, et al (2014) BRCA1 establishes DNA damage signaling and pericentric heterochromatin of the X chromosome in male meiosis. J Cell Biol 205: 663–675. 10.1083/jcb.201311050 24914237 PMC4050732

[39] Xu X, Aprelikova O, Moens P, Deng CX, Furth PA (2003) Impaired meiotic DNA-damage repair and lack of crossing-over during spermatogenesis in BRCA1 full-length isoform deficient mice. Development 130: 2001–2012. 12642502 10.1242/dev.00410

[40] Klein HL, Symington LS (2012) Sgs1—the maestro of recombination. Cell 149: 257–259. 10.1016/j.cell.2012.03.020 22500794

[41] Wicky C, Alpi A, Passannante M, Rose A, Gartner A, et al (2004) Multiple genetic pathways involving the Caenorhabditis elegans Bloom's syndrome genes him-6, rad-51, and top-3 are needed to maintain genome stability in the germ line. Mol Cell Biol 24: 5016–5027. 15143192 10.1128/MCB.24.11.5016-5027.2004 PMC416432

[42] Grabowski MM, Svrzikapa N, Tissenbaum HA (2005) Bloom syndrome ortholog HIM-6 maintains genomic stability in C. elegans. Mech Ageing Dev 126: 1314–1321. 16181657 10.1016/j.mad.2005.08.005

[43] Bickel JS, Chen L, Hayward J, Yeap SL, Alkers AE, et al (2010) Structural maintenance of chromosomes (SMC) proteins promote homolog-independent recombination repair in meiosis crucial for germ cell genomic stability. PLoS Genet 6: e1001028 10.1371/journal.pgen.1001028 20661436 PMC2908675

[44] Alpi A, Pasierbek P, Gartner A, Loidl J (2003) Genetic and cytological characterization of the recombination protein RAD-51 in Caenorhabditis elegans. Chromosoma 112: 6–16. 12684824 10.1007/s00412-003-0237-5

[45] Smolikov S, Eizinger A, Hurlburt A, Rogers E, Villeneuve AM, et al (2007) Synapsis-defective mutants reveal a correlation between chromosome conformation and the mode of double-strand break repair during Caenorhabditis elegans meiosis. Genetics 176: 2027–2033. 17565963 10.1534/genetics.107.076968 PMC1950611

[46] Severson AF, Ling L, van Zuylen V, Meyer BJ (2009) The axial element protein HTP-3 promotes cohesin loading and meiotic axis assembly in C. elegans to implement the meiotic program of chromosome segregation. Genes Dev 23: 1763–1778. 10.1101/gad.1808809 19574299 PMC2720254

[47] MacQueen AJ, Colaiacovo MP, McDonald K, Villeneuve AM (2002) Synapsis-dependent and -independent mechanisms stabilize homolog pairing during meiotic prophase in C. elegans. Genes Dev 16: 2428–2442. 12231631 10.1101/gad.1011602 PMC187442

[48] Bhalla N, Wynne DJ, Jantsch V, Dernburg AF (2008) ZHP-3 acts at crossovers to couple meiotic recombination with synaptonemal complex disassembly and bivalent formation in C. elegans. PLoS Genet 4: e1000235 10.1371/journal.pgen.1000235 18949042 PMC2567099

[49] Yokoo R, Zawadzki KA, Nabeshima K, Drake M, Arur S, et al (2012) COSA-1 reveals robust homeostasis and separable licensing and reinforcement steps governing meiotic crossovers. Cell 149: 75–87. 10.1016/j.cell.2012.01.052 22464324 PMC3339199

[50] Hillers KJ, Villeneuve AM (2009) Analysis of meiotic recombination in Caenorhabditis elegans. Methods Mol Biol 557: 77–97. 10.1007/978-1-59745-527-5_7 19799178

[51] Davis MW, Hammarlund M, Harrach T, Hullett P, Olsen S, et al (2005) Rapid single nucleotide polymorphism mapping in C. elegans. BMC Genomics 6: 118 16156901 10.1186/1471-2164-6-118 PMC1242227

[52] Rockman MV, Kruglyak L (2009) Recombinational landscape and population genomics of Caenorhabditis elegans. PLoS Genet 5: e1000419 10.1371/journal.pgen.1000419 19283065 PMC2652117

[53] Rose AM, Baillie DL (1979) A mutation in Caenorhabditis elegans that increases recombination frequency more than threefold. Nature 281: 599–600. 492325 10.1038/281599a0

[54] Schvarzstein M, Pattabiraman D, Libuda DE, Ramadugu A, Tam A, et al (2014) DNA helicase HIM-6/BLM both promotes MutSgamma-dependent crossovers and antagonizes MutSgamma-independent interhomolog associations during caenorhabditis elegans meiosis. Genetics 198: 193–207. 10.1534/genetics.114.161513 25053665 PMC4174932

[55] Zetka MC, Rose AM (1995) Mutant rec-1 eliminates the meiotic pattern of crossing over in Caenorhabditis elegans. Genetics 141: 1339–1349. 8601478 10.1093/genetics/141.4.1339 PMC1206871

[56] Jagut M, Hamminger P, Woglar A, Millonigg S, Paulin L, Mikl M, et al (2016) Separable roles for a C. elegans RMI1 homolog in promoting and antagonizing meiotic crossovers ensure faithful chromosome inheritance. PLoS Biol 14(3): e1002412. 27011106 10.1371/journal.pbio.1002412 PMC4807110

[57] Nabeshima K, Villeneuve AM, Colaiacovo MP (2005) Crossing over is coupled to late meiotic prophase bivalent differentiation through asymmetric disassembly of the SC. J Cell Biol 168: 683–689. 15738262 10.1083/jcb.200410144 PMC2171829

[58] Zetka MC, Kawasaki I, Strome S, Muller F (1999) Synapsis and chiasma formation in Caenorhabditis elegans require HIM-3, a meiotic chromosome core component that functions in chromosome segregation. Genes Dev 13: 2258–2270. 10485848 10.1101/gad.13.17.2258 PMC317003

[59] Matos J, Blanco MG, West SC (2013) Cell-cycle kinases coordinate the resolution of recombination intermediates with chromosome segregation. Cell Rep 4: 76–86. 10.1016/j.celrep.2013.05.039 23810555

[60] Torres-Rosell J, Machin F, Farmer S, Jarmuz A, Eydmann T, et al (2005) SMC5 and SMC6 genes are required for the segregation of repetitive chromosome regions. Nat Cell Biol 7: 412–419. 15793567 10.1038/ncb1239

[61] Cheok CF, Bachrati CZ, Chan KL, Ralf C, Wu L, et al (2005) Roles of the Bloom's syndrome helicase in the maintenance of genome stability. Biochem Soc Trans 33: 1456–1459. 16246145 10.1042/BST0331456

[62] Shrivastav M, De Haro LP, Nickoloff JA (2008) Regulation of DNA double-strand break repair pathway choice. Cell Res 18: 134–147. 18157161 10.1038/cr.2007.111

[63] Lemmens BB, Johnson NM, Tijsterman M (2013) COM-1 promotes homologous recombination during Caenorhabditis elegans meiosis by antagonizing Ku-mediated non-homologous end joining. PLoS Genet 9: e1003276 10.1371/journal.pgen.1003276 23408909 PMC3567172

[64] Meier B, Cooke SL, Weiss J, Bailly AP, Alexandrov LB, et al (2014) C. elegans whole-genome sequencing reveals mutational signatures related to carcinogens and DNA repair deficiency. Genome Res 24: 1624–1636. 10.1101/gr.175547.114 25030888 PMC4199376

[65] Lieber MR (2010) The mechanism of double-strand DNA break repair by the nonhomologous DNA end-joining pathway. Annu Rev Biochem 79: 181–211. 10.1146/annurev.biochem.052308.093131 20192759 PMC3079308

[66] Wolters S, Ermolaeva MA, Bickel JS, Fingerhut JM, Khanikar J, et al (2014) Loss of Caenorhabditis elegans BRCA1 promotes genome stability during replication in smc-5 mutants. Genetics 196: 985–999. 10.1534/genetics.113.158295 24424777 PMC3982690

[67] Zickler D, Kleckner N (1999) Meiotic chromosomes: integrating structure and function. Annu Rev Genet 33: 603–754. 10690419 10.1146/annurev.genet.33.1.603

[68] Hirano T (2005) Condensins: organizing and segregating the genome. Curr Biol 15: R265–275. 15823530 10.1016/j.cub.2005.03.037

[69] Stear JH, Roth MB (2002) Characterization of HCP-6, a C. elegans protein required to prevent chromosome twisting and merotelic attachment. Genes Dev 16: 1498–1508. 12080088 10.1101/gad.989102 PMC186334

[70] Chan RC, Severson AF, Meyer BJ (2004) Condensin restructures chromosomes in preparation for meiotic divisions. J Cell Biol 167: 613–625. 15557118 10.1083/jcb.200408061 PMC2172564

[71] Serrentino ME, Borde V (2012) The spatial regulation of meiotic recombination hotspots: are all DSB hotspots crossover hotspots? Exp cell Res 318: 1347–1352. 10.1016/j.yexcr.2012.03.025 22487095

[72] Terasawa M, Shinohara A, Hotta Y, Ogawa H, Ogawa T (1995) Localization of RecA-like recombination proteins on chromosomes of the lily at various meiotic stages. Genes Dev 9: 925–934. 7774810 10.1101/gad.9.8.925

[73] Moens PB, Chen DJ, Shen Z, Kolas N, Tarsounas M, et al (1997) Rad51 immunocytology in rat and mouse spermatocytes and oocytes. Chromosoma 106: 207–215. 9254722 10.1007/s004120050241

[74] Franklin AE, McElver J, Sunjevaric I, Rothstein R, Bowen B, et al (1999) Three-dimensional microscopy of the Rad51 recombination protein during meiotic prophase. Plant Cell 11: 809–824. 10330467 10.1105/tpc.11.5.809 PMC144225

[75] Buhler C, Borde V, Lichten M (2007) Mapping meiotic single-strand DNA reveals a new landscape of DNA double-strand breaks in Saccharomyces cerevisiae. PLoS Biol 5: e324 10.1371/journal.pbio.0060104 18076285 PMC2121111

[76] Lemmens BB, Tijsterman M (2011) DNA double-strand break repair in Caenorhabditis elegans. Chromosoma 120: 1–21. 10.1007/s00412-010-0296-3 21052706 PMC3028100

[77] Rockmill B, Fung JC, Branda SS, Roeder GS (2003) The Sgs1 helicase regulates chromosome synapsis and meiotic crossing over. Curr Biol 13: 1954–1962. 14614820 10.1016/j.cub.2003.10.059

[78] Cromie GA, Hyppa RW, Smith GR (2008) The fission yeast BLM homolog Rqh1 promotes meiotic recombination. Genetics 179: 1157–1167. 10.1534/genetics.108.088955 18562672 PMC2475723

[79] McVey M, Andersen SL, Broze Y, Sekelsky J (2007) Multiple functions of Drosophila BLM helicase in maintenance of genome stability. Genetics 176: 1979–1992. 17507683 10.1534/genetics.106.070052 PMC1950607

[80] Moens PB, Freire R, Tarsounas M, Spyropoulos B, Jackson SP (2000) Expression and nuclear localization of BLM, a chromosome stability protein mutated in Bloom's syndrome, suggest a role in recombination during meiotic prophase. J Cell Sci 113 (Pt 4): 663–672. 10652259 10.1242/jcs.113.4.663

[81] Croteau DL, Popuri V, Opresko PL, Bohr VA (2014) Human RecQ helicases in DNA repair, recombination, and replication. Annu Rev Biochem 83: 519–552. 10.1146/annurev-biochem-060713-035428 24606147 PMC4586249

[82] Manthei KA, Keck JL (2013) The BLM dissolvasome in DNA replication and repair. Cellular and molecular life sciences: CMLS 70: 4067–4084. 10.1007/s00018-013-1325-1 23543275 PMC3731382

[83] Wagner CR, Kuervers L, Baillie DL, Yanowitz JL (2010) xnd-1 regulates the global recombination landscape in Caenorhabditis elegans. Nature 467: 839–843. 10.1038/nature09429 20944745 PMC3045774

[84] Saito TT, Mohideen F, Meyer K, Harper JW, Colaiacovo MP (2012) SLX-1 is required for maintaining genomic integrity and promoting meiotic noncrossovers in the Caenorhabditis elegans germline. PLoS Genet 8: e1002888 10.1371/journal.pgen.1002888 22927825 PMC3426554

[85] Meneely PM, McGovern OL, Heinis FI, Yanowitz JL (2012) Crossover distribution and frequency are regulated by him-5 in Caenorhabditis elegans. Genetics 190: 1251–1266. 10.1534/genetics.111.137463 22267496 PMC3316641

[86] Chung G, Rose AM, Petalcorin MI, Martin JS, Kessler Z, et al (2015) REC-1 and HIM-5 distribute meiotic crossovers and function redundantly in meiotic double-strand break formation in Caenorhabditis elegans. Genes Dev 29: 1969–1979. 10.1101/gad.266056.115 26385965 PMC4579353

[87] Gallego-Paez LM, Tanaka H, Bando M, Takahashi M, Nozaki N, et al (2014) Smc5/6-mediated regulation of replication progression contributes to chromosome assembly during mitosis in human cells. Mol Biol Cell 25: 302–317. 10.1091/mbc.E13-01-0020 24258023 PMC3890350

[88] Tsai CJ, Mets DG, Albrecht MR, Nix P, Chan A, et al (2008) Meiotic crossover number and distribution are regulated by a dosage compensation protein that resembles a condensin subunit. Genes Dev 22: 194–211. 10.1101/gad.1618508 18198337 PMC2192754

[89] Mets DG, Meyer BJ (2009) Condensins regulate meiotic DNA break distribution, thus crossover frequency, by controlling chromosome structure. Cell 139: 73–86. 10.1016/j.cell.2009.07.035 19781752 PMC2785808

[90] Rinaldo C, Bazzicalupo P, Ederle S, Hilliard M, La Volpe A (2002) Roles for Caenorhabditis elegans rad-51 in meiosis and in resistance to ionizing radiation during development. Genetics 160: 471–479. 11861554 10.1093/genetics/160.2.471 PMC1461995

[91] Oh SD, Lao JP, Hwang PY, Taylor AF, Smith GR, et al (2007) BLM ortholog, Sgs1, prevents aberrant crossing-over by suppressing formation of multichromatid joint molecules. Cell 130: 259–272. 17662941 10.1016/j.cell.2007.05.035 PMC2034285

[92] Kaur H, De Muyt A, Lichten M (2015) Top3-Rmi1 DNA single-strand decatenase is integral to the formation and resolution of meiotic recombination intermediates. Mol Cell 57: 583–594. 10.1016/j.molcel.2015.01.020 25699707 PMC4338413

[93] Timmons L, Court DL, Fire A (2001) Ingestion of bacterially expressed dsRNAs can produce specific and potent genetic interference in Caenorhabditis elegans. Gene 263: 103–112. 11223248 10.1016/s0378-1119(00)00579-5

