# Supplementary material for: The SMC-5/6 Complex and the HIM-6 (BLM) Helicase Synergistically Promote Meiotic Recombination Intermediate Processing and Chromosome Maturation during Caenorhabditis elegans Meiosis
Source: PLoS Genet. 2016 Mar 24;12(3):e1005872. doi: 10.1371/journal.pgen.1005872 (PMC4807058; doi:10.1371/journal.pgen.1005872)
Supplement: S1 Table — (DOCX) [file pgen.1005872.s007.docx]

**Supplementary Table S1. List of strains used in this study.**

| **Strain name** | **Genotype** |
| --- | --- |
| YE57 | *smc-5(ok2421)/mIn1 [mIs14 dpy-10(e128)] II* |
| YE59 | *smc-5(tm2868)/mIn1 [mIs14 dpy-10(e128)] II* |
| YE58 | *smc-6(ok3294)/mIn1 [mIs14 dpy-10(e128)] II* |
| TG1815 | *him-6(ok412) IV* |
| CB1479 | *him-6(e1423) IV* |
| TG1860 | *him-6(e1104) IV* |
| AV157 | *spo-11(me44)/nT1[unc-?(n754) let-? qIs50] IV* |
| DW102 | *brc-1(tm1145) III* |
| VC655 | *brd-1(gk297) III* |
| YA665 | *lig-4(ok716) III* |
| CB5584 | *mIS12 II, described in wormbase* |
| UV7 | *unc-119(ed3) III; jfIs2 [pie-promoter::GFP::zhp-3 + unc-119(+)]* |
| TG2512 | *gtIs2512[Ppie-1::his-11::GFP]* |
| TG3930 | *smc-5(ok2421) smc-6(ok3294)/mIn1 II* |
| TG2578 | *smc-5(ok2421)/mIn1 II; him-6(ok412) IV* |
| TG3939 | *smc-5(ok2421)/mIn1 II; him-6(e1423) IV* |
| TG2695 | *smc-5(ok2421)/mIn1 II; him-6(e1104) IV* |
| TG2692 | *smc-6(ok3294)/mIn1 II; him-6(ok412) IV* |
| TG2693 | *smc-5(tm2868)/mIn1 II; him-6(ok412) IV* |
| TG3940 | *smc-5(ok2421)/mIn1 II; Hawaii V* |
| TG2366 | *him-6(ok412) IV; Hawaii V* |
| TG2926 | *smc-5(ok2421)/mIn1 II; him-6(ok412) IV; Hawaii V* |
| TG3941 | *him-6(ok412) IV; unc-119(ed3) III?; jfIs2 [pie-promoter::GFP::zhp-3 + unc-119(+)]* |
| TG3515 | *smc-5(ok2421)/mIn1 II; unc-119(ed3) III?; jfIs2 [pie-promoter::GFP::zhp-3 + unc-119(+)]* |
| TG3942 | *smc-5(ok2421)/mIn1 II; him-6(ok412) IV; unc-119(ed3) III?; jfIs2 [pie-promoter::GFP::zhp-3 + unc-119(+)]* |
| TG3952 | *smc-5(ok2421)/mIn1 II; him-6(ok412) IV spo-11(me44)/nT1 IV* |
| TG2429 | *him-6(ok412) IV; unc-119(ed3) III?; gtIs2512[Ppie-1::his-11::GFP]* |
| TG2643 | *smc-5(ok2421)/mIn1 II; unc-119(ed3) III ?; gtIs2512[Ppie-1::his-11::GFP]* |
| TG3944 | *smc-5(ok2421)/mIn1 II; him-6(ok412) IV; unc-119(ed3) III?; gtIs2512[Ppie-1::his-11::GFP]* |
| TG2697 | *smc-5(ok2421)/mIn1 II; brc-1(tm1145) III* |
| TG3945 | *brc-1(tm1145) III; him-6(ok412) IV* |
| TG3946 | *smc-5(ok2421)/mIn1 II; brc-1(tm1145) III; him-6(ok412) IV* |
| TG3947 | *smc-6(ok3294)/mIn1 II; brc-1(tm1145) III; him-6(ok412) IV* |
| TG3948 | *smc-5(ok2421)/mIn1 II; brd-1(gk297) III; him-6(ok412) IV* |
| TG3953 | *smc-5(tm2868)/mIn1 II; lig-4(ok716) III; him-6(ok412) IV* |
